# Molecular Evolutionary Analyses of the RNA-Dependent RNA Polymerase Region in Norovirus Genogroup II

**DOI:** 10.3389/fmicb.2018.03070

**Published:** 2018-12-18

**Authors:** Keita Ozaki, Yuki Matsushima, Koo Nagasawa, Takumi Motoya, Akihide Ryo, Makoto Kuroda, Kazuhiko Katayama, Hirokazu Kimura

**Affiliations:** ^1^Graduate School of Health Sciences, Gunma Paz University, Takasaki, Japan; ^2^Niitaka Co., Ltd., Osaka, Japan; ^3^Division of Virology, Kawasaki City Institute for Public Health, Kawasaki, Japan; ^4^Department of Pediatrics, Graduate School of Medicine, Chiba University, Chiba, Japan; ^5^Ibaraki Prefectural Institute of Public Health, Mito, Japan; ^6^Department of Microbiology, Yokohama City University School of Medicine, Yokohama, Japan; ^7^Pathogen Genomics Center, National Institute of Infectious Diseases, Tokyo, Japan; ^8^Laboratory of Viral Infection I, Kitasato Institute for Life Sciences Graduate School of Infection Control Sciences, Kitasato University, Tokyo, Japan

**Keywords:** molecular evolution, norovirus, GII, bioinformatics, RNA-dependent RNA polymerase, negative selection

## Abstract

Noroviruses are the leading cause of viral gastroenteritis in humans across the world. RNA-dependent RNA polymerase (RdRp) plays a critical role in the replication of the viral genome. Although there have been some reports on a limited number of genotypes with respect to the norovirus evolution of the *RdRp* region, no comprehensive molecular evolution examination of the norovirus GII genotype has yet been undertaken. Therefore, we conducted an evolutionary analysis of the 25 genotypes of the norovirus GII *RdRp* region (full-length), collected globally using different bioinformatics technologies. The time-scaled phylogenetic tree, generated using the Bayesian Markov Chain Monte Carlo (MCMC) method, indicated that the common ancestor of GII diverged from GIV around 1443 CE [95% highest posterior density (HPD), 1336–1542]. The GII *RdRp* region emerged around 1731 CE (95% HPD, 1703–1757), forming three lineages. The evolutionary rate of the *RdRp* region of the norovirus GII strains was estimated at over 10^−3^ substitutions/site/year. The evolutionary rates were significantly distinct in each genotype. The composition of the phylogenetic distances differed among the strains for each genotype. Furthermore, we mapped the negative selection sites on the RdRp protein and many of these were predicted in the GII.P4 RdRp proteins. The phylodynamics of GII.P4, GII.P12, GII.P16, and GII.Pe showed that their effective population sizes increased during the period from 2003 to 2014. Our results cumulatively suggest that the *RdRp* region of the norovirus GII rapidly and uniquely evolved with a high divergence similar to that of the norovirus *VP1* gene.

## Introduction

Noroviruses belonging to the family *Caliciviridae*, genus *Norovirus*, are a major causative agent of gastroenteritis (Green, 2013; Robilotti et al., 2015). Accumulating epidemiological evidence suggests that norovirus infections affect both children and adults and that, globally, this agent causes approximately 60% of all viral gastroenteritis cases and 200,000 deaths in children under 5 years old (Robilotti et al., 2015). Moreover, noroviruses cause large-scale food poisoning outbreaks throughout the world (Le Guyader et al., 2008; Räsänen et al., 2010; Hardstaff et al., 2018).

Noroviruses are classified into seven genogroups (GI–GVII) according to the norovirus *VP1* gene sequences (Vinjé, 2015). Among them, GI, GII and GIV viruses are known to infect humans. Furthermore, GI and GII viruses are classified into 9 and 22 genotypes, respectively, by their *VP1* gene sequences (Vinjé, 2015). The norovirus genome encodes three open reading frames (ORF). Of these, ORF1 encodes seven genes, including that of the RNA-dependent RNA polymerase (*RdRp*) region. The *RdRp* region plays a critical role in the norovirus genome replication.

Previous reports have suggested that the GII.P2 and GII.P16 *RdRp* regions exhibit a large genetic divergence as does the *VP1* gene encoded in ORF2 (Nagasawa et al., 2018a). The RdRp genotypes of GII viruses are currently classified into nearly 30 genotypes on the basis of their *RdRp* region sequences (Vinjé, 2015; Chhabra et al., 2018). Furthermore, recombination of the norovirus genome frequently occurs at ORF1 and at two junctions, resulting in the formation of new chimeric viruses (Bull et al., 2005; Bull et al., 2007). For example, the ORF1 of the GII.2 virus primarily encodes two distinct types of RdRp proteins, which are produced by GII.P2 and GII.P16 (GII.P2–GII.2 and GII.P16–GII.2, a chimeric virus; Bidalot et al., 2017; Lu et al., 2017; Mizukoshi et al., 2017; Niendorf et al., 2017). Notably, a previous report revealed the *VP1* gene of the GII.2 virus encoding two distinct *RdRp* genotypes reside in different clusters (Mizukoshi et al., 2017). Furthermore, the evolutionary rate of the *VP1* gene in these viruses differs significantly (Mizukoshi et al., 2017). Thus, it is possible that the *RdRp* region modulates the evolution of the *VP1* gene (Mizukoshi et al., 2017). These observations imply that it is important to study the evolution of the *RdRp* region of GII viruses because little information is presently available. Moreover, GII viruses representing multiple genotypes may represent the major causative agents of gastroenteritis.

Recently developed bioinformatics methods have enabled the analysis of the evolution of various viral genes, including those of the norovirus (Kimura et al., 2015; Kobayashi et al., 2015; Takahashi et al., 2018). Here, we conducted a detailed molecular evolutionary analysis of the *RdRp* region in the norovirus GII viruses on the basis of the globally collected strain sequences.

## Materials and Methods

### Strain Selection

Full-length nucleotide sequences of the norovirus GII *RdRp* region were collected from the GenBank^1^ (accessed on May 10, 2018). The classification of the strains was conducted using a norovirus genotyping tool (Kroneman et al., 2011) and all the sequences of the GII genogroup were selected. Strains with an unknown collection year and ambiguous sequences with undetermined base sequences (e.g., N, Y, and V) were omitted from the dataset. At this point, the dataset was comprised of approximately 1,500 strains of the *RdRp* region sequences. However, a selective pressure analysis could not be performed owing to software capacity limitations. The nucleotide identity among the 1,500 *RdRp* region sequences was calculated using Clustal Omega (Sievers et al., 2011). One sequence was randomly selected from a group of homologous sequences with ≥99.4% identities while the others were excluded from the dataset. In addition, the dataset was subjected to a protocol for the detection of recombination within the *RdRp* region using RDP4.95 software with seven primary exploratory recombination signal detection methods (RDP, GENECONV, BOOTSCAN/RESCAN, MAXCHI, CHIMAERA, SISCAN, and 3SEQ; Martin et al., 2015). The threshold of the *p*-value for significance was set to 0.001. The recombinant regions were defined as those that were detected by more than four of these methods, which were then removed from the dataset. Upon completing this refinement, 484 strains were finally used in this study (Supplementary Table S1). The sequences in the dataset were aligned with the MAFFT software (Katoh and Standley, 2013).

### Construction of a Time-Scaled Phylogenetic Tree Using the Bayesian Markov Chain Monte Carlo (MCMC) Method

Time-scaled phylogenies were constructed by the Bayesian MCMC method in the BEAST software package v2.4.8 (Drummond and Rambaut, 2007; Bouckaert et al., 2014). In order to estimate the phylogenetic relationships in the *RdRp* region between distinct norovirus genogroups, we added the nucleotide sequences of the human norovirus GI, porcine GII (GII.P11 and GII.P18), bovine GIII and human GIV strains to the dataset (yielding a total of 489 strains). First, we determined the best substitution model (GTR+I+Γ) using the jModelTest2 software (Guindon and Gascuel, 2003; Darriba et al., 2012). Next, the best of four clock models (i.e., strict clock, relaxed clock exponential, relaxed clock log normal and random local clock) and two tree prior models (i.e., coalescent constant population and coalescent exponential population) was selected by path sampling/stepping stone-sampling marginal-likelihood estimation (Baele et al., 2012). As a result, the dataset was analyzed using a strict clock and tree prior of coalescent constant population. The MCMC was run on chain lengths of 100,000,000 steps with sampling at every 2,000 steps. The analyzed data were then evaluated by the effective sample size using Tracer^2^ software and values larger than 200 were accepted. The maximum clade credibility trees were created by discarding the first 10% of the trees (burn-in) using the TreeAnnotator v2.4.8 in the BEAST2 package. The time-scaled phylogenetic trees were visualized using the FigTree^3^ v1.4.0 software. The reliability of branches was supported by the 95% HPD interval. Moreover, the evolutionary rates for the *RdRp* genotypes of the norovirus GII, including more than 10 strains (GII.P4, P7, P12, P16, P21, and Pe), were also estimated using the appropriate models determined from the datasets described above.

### Calculation of Phylogenetic Distance

Phylogenetic trees were created from the datasets of the norovirus GII genotypes and each *RdRp* genotype, including more than 10 strains, based on the maximum likelihood (ML) method in the MEGA7 software package (Kumar et al., 2016). The best substitution models were determined using the jModelTest2. The phylogenetic distances between the norovirus GII strains were calculated from the pairwise ML distance of the ML tree using Patristic software (Fourment and Gibbs, 2006).

### Construction of Three-Dimensional Structures and Selective Pressure Analyses

The structural models of the RdRp proteins (GII.P1:U07611, GII.P2:DQ456824, GII.P3:KJ194500, GII.P4:AB541272, GII.P5:KJ196288, GII.P6:AB039778, GII.P7:AB039777, GII.P12:AB220922, GII.P15:KU954108, GII.P16:KJ196286, GII.P17:LC037415, GII.P20:EU424333, GII.P21:AY919139, GII.P22:KJ196277, GII.P23:MG495080, GII.P24:KY225989, GII.Pc:AY134748, GII.Pe:JX459907, GII.Pf:MF405169, GII.Pg:GQ845370, GII.Pj:KC576911, and GII.Pm:KJ194507) were constructed using the homology modeling MODELLER v9.20 (Webb and Sali, 2014, 2016). The crystal structures of the GII.P4 strain RdRp protein (PDB ID: 1SH0) were used as the template for homology modeling. The amino acid sequences of the template and target strains were aligned using the MAFFTash software (Standley et al., 2007; Katoh et al., 2009). The constructed structures were minimized using GROMOS96 (van Gunsteren et al., 1996) implemented in the Swiss PDB Viewer v4.1 (Guex and Peitsch, 1997) and structural reliability was evaluated using Ramachandran plots through the RAMPAGE server (Lovell et al., 2003), resulting in favored regions of 97.7 ± 0.44%, allowed regions of 1.9 ± 0.43% and outlier regions of 0.4 ± 0.09% [mean ± standard deviation (SD)] for all residues in each structure. Non-synonymous (dN) and synonymous (dS) substitution rates at each codon were calculated using the Datamonkey server to estimate the positive and negative selection sites in the norovirus GII *RdRp* regions and in each genotype containing more than three strains (Pond and Frost, 2005; Delport et al., 2010). Sites under positive and negative selection were assigned by three methods [single-likelihood ancestor counting (SLAC), fixed effects likelihood (FEL), and internal fixed effects likelihood (IFEL)], using a significance level of *p* < 0.05. A two-tailed extended binomial distribution was used to calculate the *p*-value for SLAC. The FEL and IFEL were based on a single-degree-of-freedom likelihood ratio test (using an asymptotic chi-squared distribution) for classifying a site as positively or negatively selected. The final structural models were modified and colored using the Chimera v1.13 software (Pettersen et al., 2004). The substitution sites of the other RdRp proteins, compared to a prototype GII.P8 strain (accession no. AB039780) and the negative selection at these sites were mapped onto the structure.

### Bayesian Skyline Plot Analysis

The genealogical population size of the norovirus strains was estimated using the Bayesian skyline plot algorithm with BEAST v2.4.8. Appropriate substitution and clock models were selected as described above. The analyzed plots were visualized with 95% HPD using Tracer^2^.

### Statistical Analyses

Statistical analyses were conducted using EZR statistical software on the Kruskal–Wallis test with multiple comparisons and the Holm test for evolutionary rates and phylogenetic distances (Kanda, 2013). Detailed statistical data are presented in Supplementary Tables S2, S3.

## Results

### Time-Scaled Phylogenetic Tree Constructed Using the Bayesian MCMC Method

**FIGURE 1:**
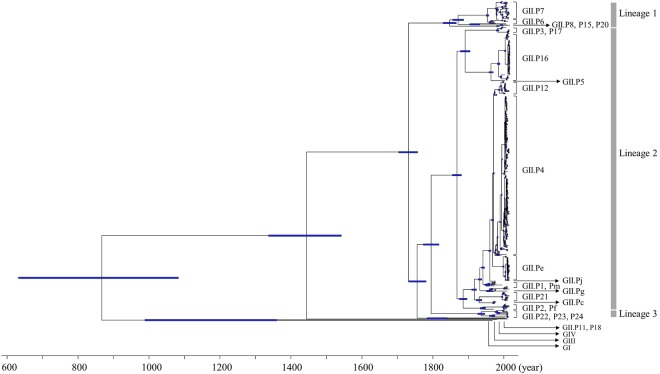
Time-scaled phylogenetic tree of the full-length norovirus *RdRp* regions constructed by the Bayesian MCMC method. The maximum clade credibility tree with a dataset including norovirus GI.P1, GII, GIII, and GIV is shown. The blue bars indicate the 95% HPD for the estimated years of common ancestors.

A time-scaled phylogenetic tree of the *RdRp* region for the norovirus GII virus was constructed using the Bayesian MCMC method (BEAST v2.4.8). The tree clearly classified the 23 genotypes of the human norovirus GII strains into three lineages by setting the cut-off value of phylogenetic distances for lineages as 1.0 (Figures 1, 3A): lineage 1 (GII.P6-P8, P15 and P20), lineage 2 (GII.P1-P5, P12, P16, P17, P21, Pc, Pe, Pf, Pg, Pj, and Pm) and lineage 3 (GII.P22, P23, and P24) (Figure 1). Notably, the P4 genotype strains formed several clusters.

The tree showed that an ancient common ancestor of GI, GII, GIII and GIV diverged in 867 CE (95% HPD, 632–1082) and that the common ancestor of GII diverged from GIV in 1443 CE (95% HPD, 1336–1542). The common ancestor of the norovirus GII viruses evolved three lineages after 1731 CE (95% HPD, 1703–1757). Lineage 1 diverged in 1847 CE (95% HPD, 1828–1866), lineage 2 diverged in 1868 CE (95% HPD, 1855–1880) and lineage 3 diverged in 1936 CE (95% HPD, 1926–1946). GII.P8 diverged from a common ancestor with the other norovirus GII virus in 1847 CE (95% HPD, 1828–1866; Supplementary Figure S1). We have shown the detailed estimated years of common ancestors, which are defined as year of divergence, for the three lineages and the genotypes including two or more strains, in Table 1.

**Table 1:** The year of divergence in each genotype of norovirus GII strains.

Genogroup	Lineage	Genotypes	Year of divergence (95% HPD)
GII	1	GII.P6, P7, P8, P15, and P20	1847 (1828–1866)
	2	GII.P1, P2, P3, P4, P5, P12, P16, P17, P21, Pc, Pe, Pf, Pg, Pj, and Pm	1868 (1855–1880)
	3	GII.P22, P23, and P24	1936 (1926–1946)
		GII.P1	1949 (1946–1953)
		GII.P2	1998 (1996–2000)
		GII.P3	1991 (1990–1993)
		GII.P4	1971 (1969–1973)
		GII.P6	1961 (1958–1963)
		GII.P7	1978 (1976–1980)
		GII.P8	1982 (1979–1984)
		GII.P12	1987 (1984–1989)
		GII.P15	1992 (1988–1996)
		GII.P16	1985 (1981–1988)
		GII.P17	2010 (2009–2011)
		GII.P21	1995 (1993–1997)
		GII.P22	1979 (1974–1983)
		GII.P23	2008 (2007–2009)
		GII.P24	2010 (2009–2011)
		GII.Pc	1970 (1968–1971)
		GII.Pe	1998 (1996–2000)
		GII.Pg	1956 (1950–1961)

*HPD, highest posterior density.*

**FIGURE 2:**
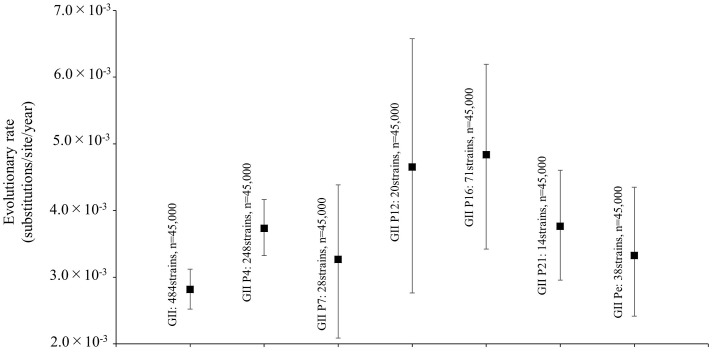
Evolutionary rates of the full-length nucleotide sequences of the norovirus *RdRp* regions. The *y*-axis represents the evolutionary rate (substitutions/site/year) and the *x*-axis represents each genotype. The black squares indicate the mean and the bars indicate the interval of 95% HPD. The statistical results for multiple comparisons between the RdRp genotypes are shown in Supplementary Table S2.

Furthermore, we also analyzed the evolutionary rates of the norovirus GII strains and each polymerase genotype. The evolutionary rate of the *RdRp* region in the norovirus GII strains was estimated at 2.82 × 10^−3^ substitutions/site/year (95% HPD, 2.52–3.12 × 10^−3^ substitutions/site/year; Figure 2). Moreover, the nucleotide substitution rates were significantly different between the GII strains (*p* < 0.001; Figure 2 and Supplementary Table S2). Interestingly, the evolutionary rates of the GII.P12 and GII.P16 strains were higher than those of other genotypes at 4.65 × 10^−3^ substitutions/site/year (95% HPD, 2.77–6.58 × 10^−3^ substitutions/site/year) and 4.83 × 10^−3^ substitutions/site/year (95% HPD, 3.42–6.19 × 10^−3^ substitutions/site/year), respectively.

### Phylogenetic Distances of the *RdRp* Region in the Norovirus GII Strains

**FIGURE 3:**
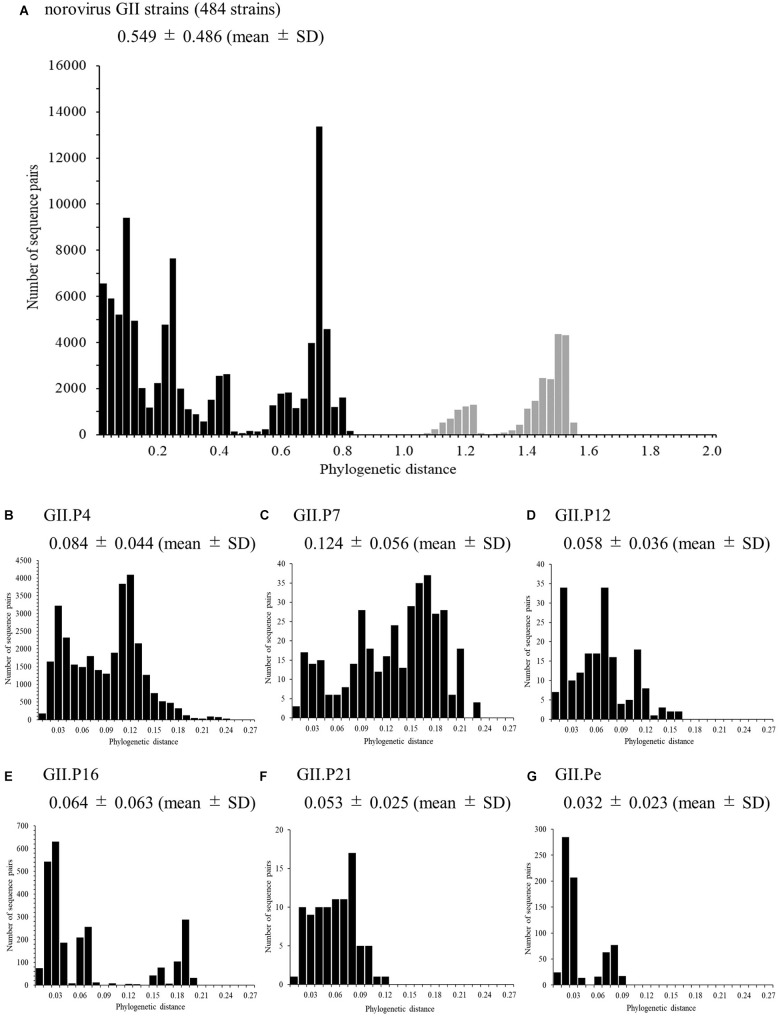
Phylogenetic distances between sequences of the full-length norovirus *RdRp* region. The distributions of phylogenetic distances of the norovirus GII strains **(A)**, GII.P4 **(B)**, GII.P7 **(C)**, GII.P12 **(D)**, GII.P16 **(E)**, GII.P21 **(F)**, and GII.Pe **(G)** are shown. The *y*-axis represents the number of sequence pairs corresponding to each distance, and the *x*-axis shows the phylogenetic distance. The distribution of phylogenetic distance for ≥1.0 is shown in gray in **(A)**. The numbers on the histograms indicate the mean ± SD of all phylogenetic distances for GII strains **(A)** and each genotype **(B–G)**. The statistical results for multiple comparisons are shown in Supplementary Table S3.

We also analyzed the phylogenetic distances of the *RdRp* region in the norovirus GII strains, which averaged 0.549 ± 0.486 (mean ± SD, Figure 3A). The phylogenetic distances of GII.P4 and GII.P7 were 0.084 ± 0.044 and 0.124 ± 0.056, respectively. These histograms were distributions with a broad range of the distances (Figures 3B,C). Furthermore, the phylogenetic distance of GII.P12 was 0.058 ± 0.036 (Figure 3D). The phylogenetic distance of GII.P16 was 0.064 ± 0.063, and the histogram was bimodal (Figure 3E). Moreover, the phylogenetic distance of GII.P21 was 0.053 ± 0.025 (Figure 3F). The phylogenetic distance of GII.Pe was 0.032 ± 0.023, and the histogram was bimodal (Figure 3G). The phylogenetic distances of each genotype differed significantly between the norovirus GII strains (*p* < 0.05 or *p* < 0.001), excluding those between GII.P12 and GII.P21. Detailed data are presented in Supplementary Table S3.

### Mapping and Structure of Amino Acid Substitutions and Negative Selection Sites on the Norovirus GII RdRp Protein

**FIGURE 4:**
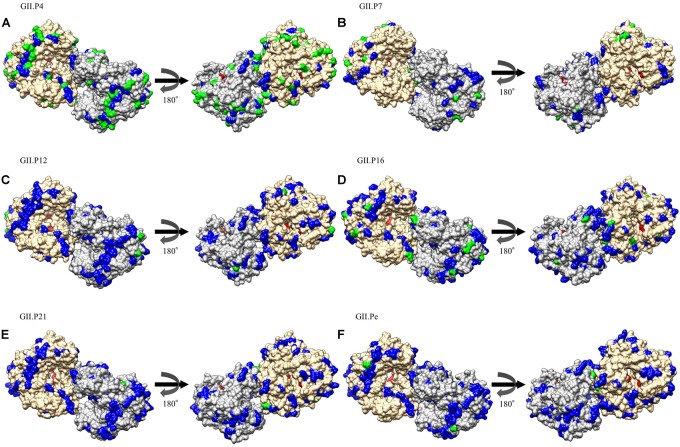
Structural models for the RdRp protein of each genotype. Three-dimensional RdRp dimer structures for GII.P4 **(A)**, GII.P7 **(B)**, GII.P12 **(C)**, GII.P16 **(D)**, GII.P21 **(E)**, and GII.Pe **(F)** are shown. The chains to composing the dimer structures are colored gray (chain A) and Navajo white (chain B). Negative selection sites are colored green. Amino acid substitutions of the other genotypes compared to the GII.P8 strain are colored blue. Active site residues are colored red.

We mapped the substitution sites of the other RdRp proteins using GII.P8 (a prototype, accession no. AB039780) as a reference strain, because GII.P8 was the first to diverge from the common ancestor of the norovirus GII polymerase region (Supplementary Figure S1). Several amino acid substitutions were predicted on the exterior structures of RdRp proteins, but no substitution was found adjacent to the active site of the enzyme (Figure 4 and Supplementary Figure S2). Five common amino acid substitutions (Met160Val, Ile221Met, Leu227Ile, Gln383Glu, and Arg408Gln) were detected in all genotypes of the RdRp protein. We first mapped the negative selection sites and amino acid substitution sites on the RdRp protein structure of the norovirus GII viruses. Of the 171 substitution sites per monomer, 158 were estimated to be negative selection sites between the prototype GII.P8 strain and other strains, according to the amino acid residues in the dataset of the norovirus GII strains (Table 2). Furthermore, of the negative selection sites in each genotype, the GII.P4, GII.P7, and GII.P16 strains contained 49, 7 and 12 sites per monomer, respectively (Table 2 and Supplementary Table S4). There were five or fewer negative selection sites per monomer in other genotypes (GII.P1, P12, P21, P22, and Pe), while no negative selection sites were found in the GII.P2, P3, P6, P17, P23, Pc, and Pg strains (Table 2 and Supplementary Table S4). No positive selection sites were predicted in all the norovirus GII strains and each genotype strain (data not shown).

**Table 2:** The number of amino acid substitutions and negative selection in the norovirus GII strains.

Genotype	Number of substitution sites^†^	Number of negative selection sites^‡^
GII.P1	101	1(1.0%)
GII.P2	92	0(0%)
GII.P3	94	0(0%)
GII.P4	97	49(50.5%)
GII.P6	39	0(0%)
GII.P7	41	7(17.1%)
GII.P12	100	5(5.0%)
GII.P16	91	12(13.2%)
GII.P17	94	0(0%)
GII.P21	94	2(2.1%)
GII.P22	107	1(0.9%)
GII.P23	99	0(0%)
GII.Pc	99	0(0%)
GII.Pe	100	2(2.0%)
GII.Pg	93	0(0%)
Norovirus GII	171	158(92.4%)

*^†^The number of substitution sites per monomer of other RdRp proteins compared to the GII.P8 strain. ^‡^The number of negative selection sites that were identified as consensus sites by the SLAC, FEL, and IFEL methods.*

### Phylodynamics of the *RdRp* Region in the Norovirus GII Strains Using the BSP Method

**FIGURE 5:**
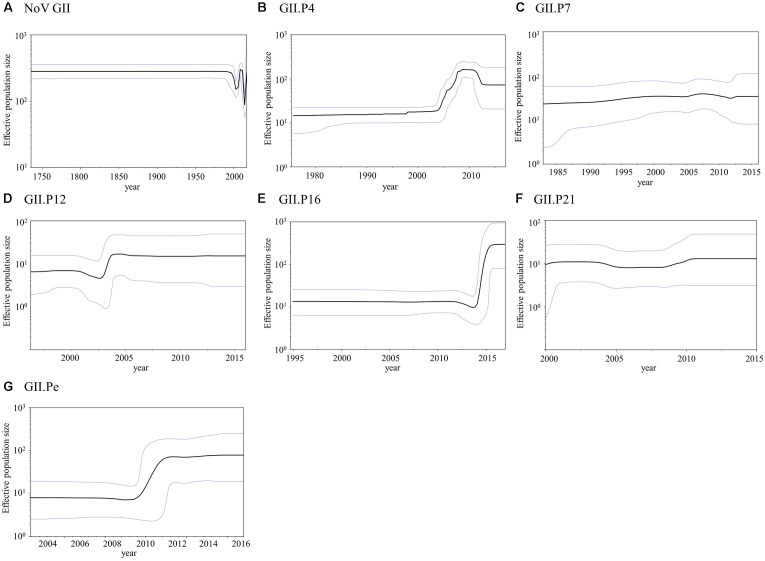
Bayesian skyline plot for the norovirus GII strains. Plots for norovirus GII **(A)**, GII.P4 **(B)**, GII.P7 **(C)**, GII.P12 **(D)**, GII.P16 **(E)**, GII.P21 **(F)**, and GII.Pe **(G)** are shown. The *y*-axis represents the effective population size on a logarithmic scale, and the *x*-axis denotes the time in years. The solid black line indicates the mean posterior value, and the blue lines indicate intervals with 95% HPD.

We analyzed the phylodynamics of the norovirus GII *RdRp* region using the BSP method; the detailed parameters are shown in Supplementary Table S5. The mean effective population size remained constant until approximately 1990 in the present norovirus GII strains, after which the values fluctuated (Figure 5A). The mean effective population size of GII.P4 increased in 2004–2008 and decreased around 2011 in each genotype (Figure 5B). The mean effective population sizes of GII.P12, GII.P16, and GII.Pe increased approximately between 2003 and 2004, 2014 and 2015, and 2009 and 2011, respectively, while no size changes were observed in the other polymerase genotypes (Figures 5C–G). In addition, the effective population sizes of the norovirus GII strains were estimated to have been maintained at over 10^2^ for approximately 250 years (Figure 5A).

## Discussion

Molecular evolutionary analyses of the norovirus *RdRp* region have been reported in some previous studies (Siebenga et al., 2010; Matsushima et al., 2015; Nagasawa et al., 2018a). For example, Siebenga et al. (2010) mainly studied the GII.P4 *RdRp* region, while Matsushima et al. (2015) studied the *RdRp* region and the *VP1* gene of GII.P17-GII.17. Furthermore, Nagasawa et al. (2018a) analyzed the *RdRp* region and the *VP1* gene of GII.P2-GII.2 and GII.P16-GII.2. To the best of our knowledge, this is the first comprehensive molecular evolutionary study of a large number of polymerase genotypes (25) from globally collected strains. Our cumulative findings suggest that (i) the common ancestor of the *RdRp* region of the analyzed strains, including GI, GII, GIII, and GIV, diverged around 1150 years ago (867 CE) and that the GII *RdRp* region diverged from GIV around 570 years ago (1443 CE) to form three major lineages of the norovirus GII strains (Figure 1); (ii) the *RdRp* region evolved rapidly (>10^−3^ substitutions/site/year) and the evolutionary rates of the different genotypes were significantly different (Figure 2); (iii) several amino acid substitutions of the RdRp protein (39–107 sites) were detected in various genotypes, while negative selection sites were distinct in each genotype (Figure 4 and Table 2); (iv) the phylodynamics of the GII *RdRp* region differed between the genotypes. These results suggest that the norovirus GII *RdRp* region evolved rapidly with several amino acid substitutions. Furthermore, the evolutionary mechanism of each genotype may be different.

The time-scaled evolutionary tree of the GII *RdRp* region using the Bayesian MCMC method showed similar evolutionary trends to the one produced using the GII *VP1* gene (Kobayashi et al., 2016). Specifically, Kobayashi et al. (2016) reported that the common ancestor of the GI, GII, GIII, and GIV *VP1* gene diverged around 1160 years ago (854 CE), while the common ancestor of the GII and GIV diverged around 570 years ago (1445 CE) and formed three major lineages of the norovirus GII. However, the divergence years of the norovirus GII *RdRp* region and the *VP1* gene were different (290 vs. 380 years ago). Although the datasets differed between the previous and present studies, time-scaled evolution of the region and the gene might fall into step.

Several evolutionary studies of the norovirus genes have been reported, although these mainly focused on the *VP1* (capsid) gene (Bok et al., 2009; Boon et al., 2011; Qiao et al., 2016; Motoya et al., 2017; Hernandez et al., 2018; Sang and Yang, 2018). However, some studies focused on the evolution of the norovirus *RdRp* region (Siebenga et al., 2010; Mahar et al., 2013; Nagasawa et al., 2018a). For example, Siebenga et al. (2010) estimated that, based on partial sequences (247 nt), the evolutionary rate of the GII.4 *RdRp* region was 8.98 × 10^−3^ substitutions/site/year. Another report showed that the rate for the GII.3 *RdRp* region (274 nt) was 2.79 × 10^−3^ substitutions/site/year (Mahar et al., 2013). Furthermore, Nagasawa et al. (2018a) estimated that the rate for the full-length GII.P16 was 2.03 × 10^−3^ substitutions/site/year. However, there have been no evolutionary studies on the various norovirus genotypes in the *RdRp* region; therefore, it was important to comprehensively study the evolution of the norovirus GII *RdRp* region from globally collected strains. In this study, we showed that the GII *RdRp* region evolved rapidly (>10^−3^ substitutions/site/year) and that distinct evolutionary rates occurred in each genotype (Figure 2). These new findings may contribute to a more complete understanding of the genetic properties of the norovirus GII *RdRp* region.

We also analyzed the phylogenetic distances in the present strains (Figure 3) and found that the composition of the distances differed among the strains for each genotype (Figures 3B–G). The histograms of the distances of the GII.P4 and GII.P7 genotypes showed broad range distributions, suggesting a larger genetic divergence than that in other genotypes. The histograms of the distances of the other genotypes showed narrow-range distributions and suggested that they were a small genetic divergence. Thus, the GII *RdRp* region exhibits a varied genetic divergence, as does the GII *VP1* gene (Kobayashi et al., 2016; Motoya et al., 2017).

We then estimated the negative selection sites in the RdRp protein *in silico* and mapped these sites. Several possible negative selection sites were identified in the RdRp protein of each genotype (Figure 4 and Supplementary Figure S2). Of these, many of the negative selection sites (49 sites per monomer) at the amino acid substitution sites, compared to the reference strain (GII.P8), were found in the GII.P4 RdRp protein. In general, negative selection sites may play a role in preventing the loss of viral protein function, because most mutations are deleterious (Domingo, 2006). In addition, other data suggest that amino acid substitutions (291Thr or 291Val) enhance the GII RdRp activity (Bull et al., 2010). The present data suggest that such substitutions occurred in the GII.P4 protein (291Thr) and that the GII.P4 may, therefore, be more efficient at replicating the viral genome, although we did not examine its activity *in vitro* in this study. Epidemiological data suggest that the GII.P4-GII.4 was responsible for the pandemics of the gastroenteritis between 2006 and 2014 (Kumazaki and Usuku, 2015). A high replication efficacy of the GII.P4 RdRp protein may have been associated with these pandemics, although further studies are required to test this hypothesis.

Finally, we also assessed the phylodynamics of various genotypes of the GII *RdRp* region (Figure 5). The phylodynamics of GII.P4, GII.P12, GII.P16, and GII.Pe fluctuated at certain times. For example, GII.P4 increased at around 2004–2008, while GII.P12, GII.P16 and GII.Pe increased at around 2003–2004, 2014–2015, and 2009–2011, respectively. We determined the year when the population size of the polymerase type increased to the year in which the VP1 genotypes of the strains were obtained. In the genotypes of GII.P4, GII.P12, and GII.Pe, the collection year of GII.4 was almost consistent with the year when the population size increased. However, in the GII.P16, the collection years of GII.2 and GII.4 agreed with the year of increase of the population size. The collection year of these detected genotypes was consistent with those of previous epidemiological reports on the GII.2 and GII.4 (Okada et al., 2006; Motomura et al., 2008; Chen et al., 2015; Nagasawa et al., 2018b). A similar phenomenon was previously reported in the phylodynamics of the GII *VP1* gene (Kobayashi et al., 2016; Sang and Yang, 2018). Therefore, information about the phylodynamics of norovirus genes may contribute to understanding the past prevalence of each norovirus genotype.

In this study, a limited number of GII RdRp sequences from the GenBank^1^ were used, which may have led to some selection bias. In addition, sequences with ≥99.4% identity were omitted. These biases may have affected the results of the bioinformatics analyses. In particular, the result of the phylogenetic distance may have been biased by analyzing the dataset of all sequences and each genotype excluding ≥99.4% identity sequences.

## Conclusion

In conclusion, the common ancestor of the GII *RdRp* region diverged around 290 years ago (1731 CE) with a high evolutionary rate (over 10^−3^ substitutions/site/year). As a result, the GII *RdRp* region and proteins exhibit high diversity. Moreover, the phylodynamics of some *RdRp* genotypes have changed drastically over the past 10 years. These results should contribute to a better understanding of the norovirus virology.

## Author Contributions

KO, KK, and HK designed the study. KO, YM, KN, TM, MK, and HK analyzed the data. KO, YM, AR, KK, and HK wrote and supervised the study. All authors read and approved the manuscript.

## Conflict of Interest Statement

KO was employed by company Niitaka Co., Ltd. The remaining authors declare that the research was conducted in the absence of any commercial or financial relationships that could be construed as a potential conflict of interest.

## Footnotes

1 http://www.ncbi.nlm.nih.gov/genbank

2 http://tree.bio.ed.ac.uk/software/tracer/

3 http://tree.bio.ed.ac.uk/software/figtree/
