# Supplementary material for: Molecular Evolutionary Analyses of the RNA-Dependent RNA Polymerase Region in Norovirus Genogroup II
Source: Front Microbiol. 2018 Dec 18;9:3070. doi: 10.3389/fmicb.2018.03070 (PMC6305289; doi:10.3389/fmicb.2018.03070)
Supplement: Supplementary file 1 [file Image_1.pdf]

Supplementary Material

Molecular Evolutionary Analyses of the RNA-Dependent RNA Polymerase Region in Norovirus Genogroup II

Keita Ozaki, Yuki Matsushima, Koo Nagasawa, Takumi Motoya, Akihide Ryo, Makoto Kuroda, Kazuhiko Katayama* and Hirokazu Kimura*

*** Correspondence:**

Prof. Hirokazu Kimura: h-kimura@paz.ac.jp

Prof. Kazuhiko Katayama: katayama@lisci.kitasato-u.ac.jp,

Supplementary Figures

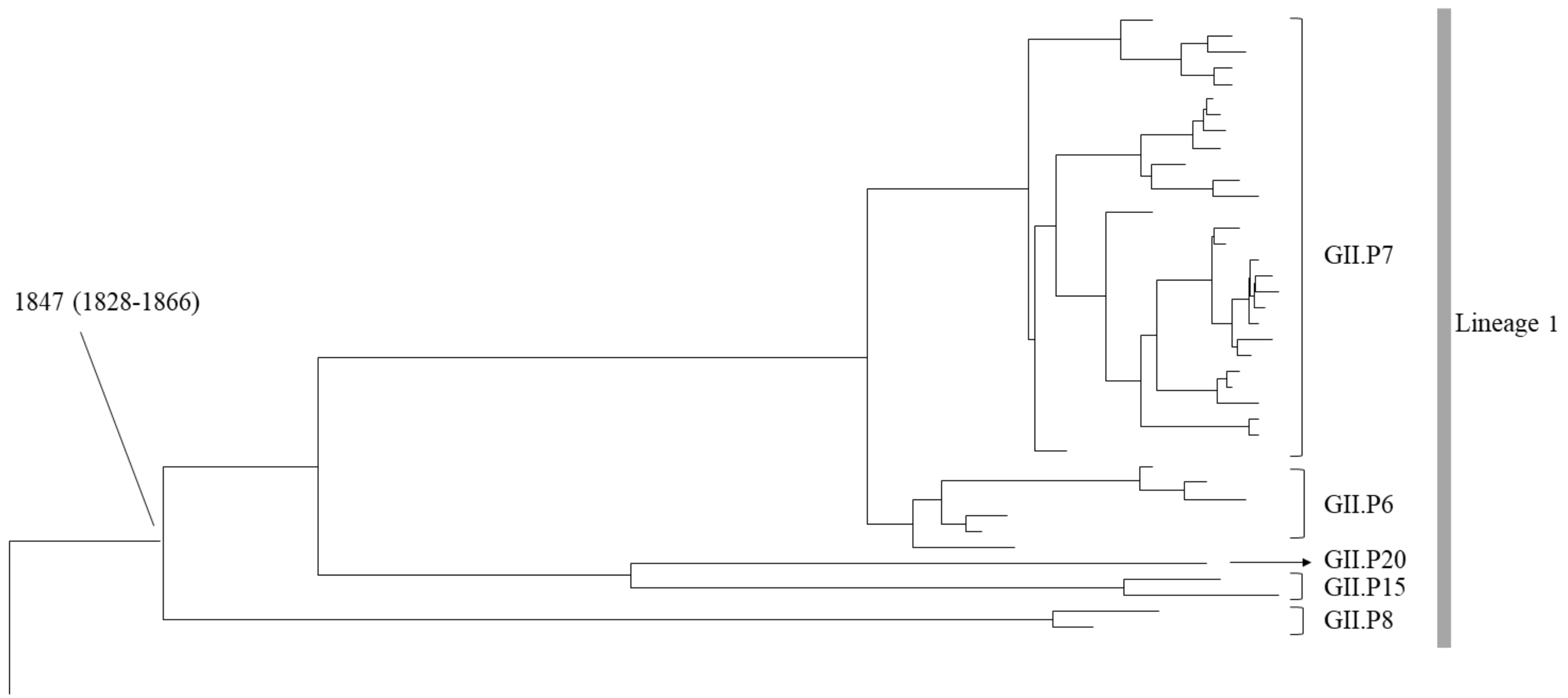

Figure S1. Time scaled phylogenetic trees of the norovirus *RdRp* region constructed by the Bayesian MCMC method. Enlarged tree focused on the lineage 1 (GII.P6, P7, P8, P15 and P20).

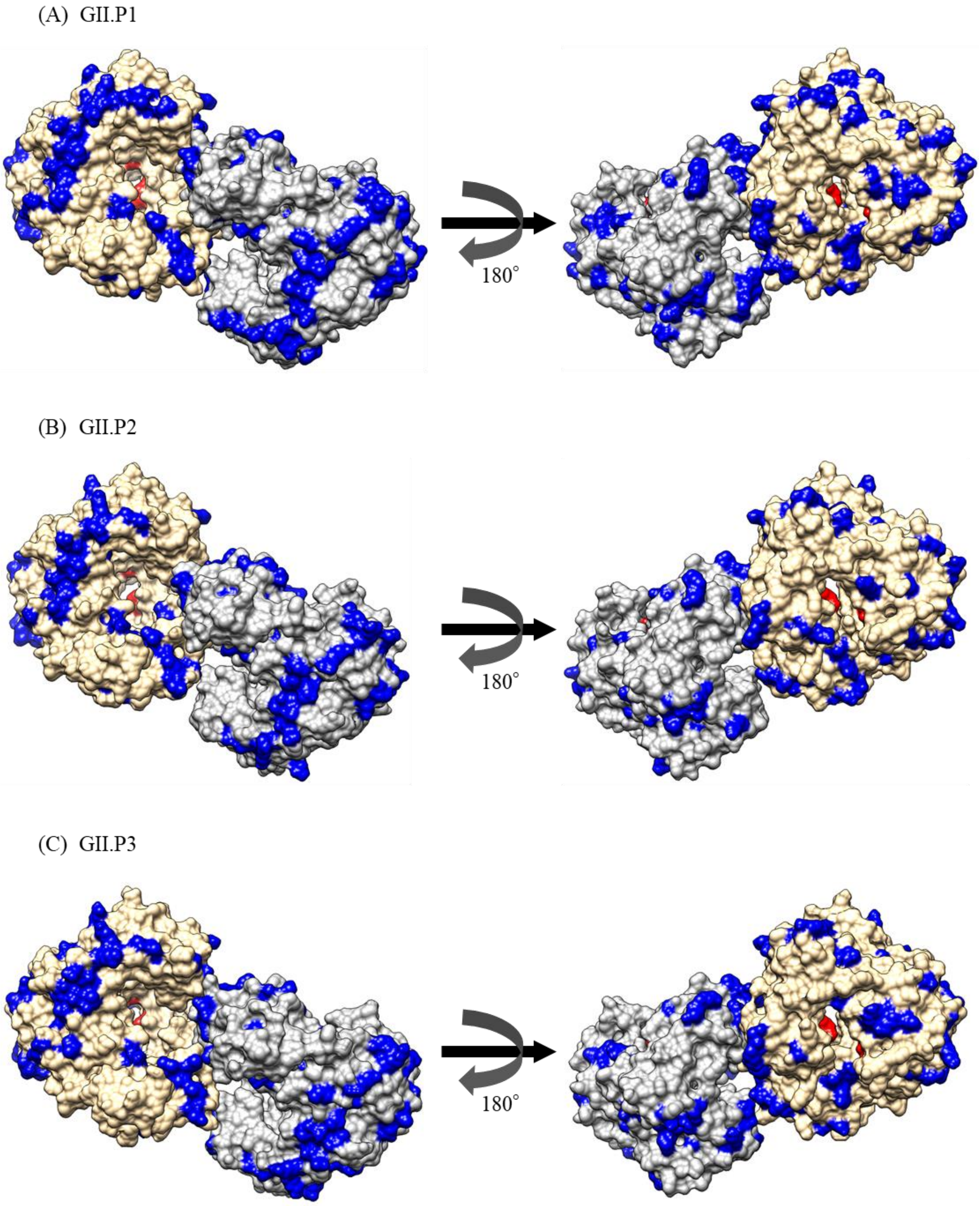
(A) GII.P1
180°
(B) GII.P2
180°
(C) GII.P3
180°

(D) GII.P5

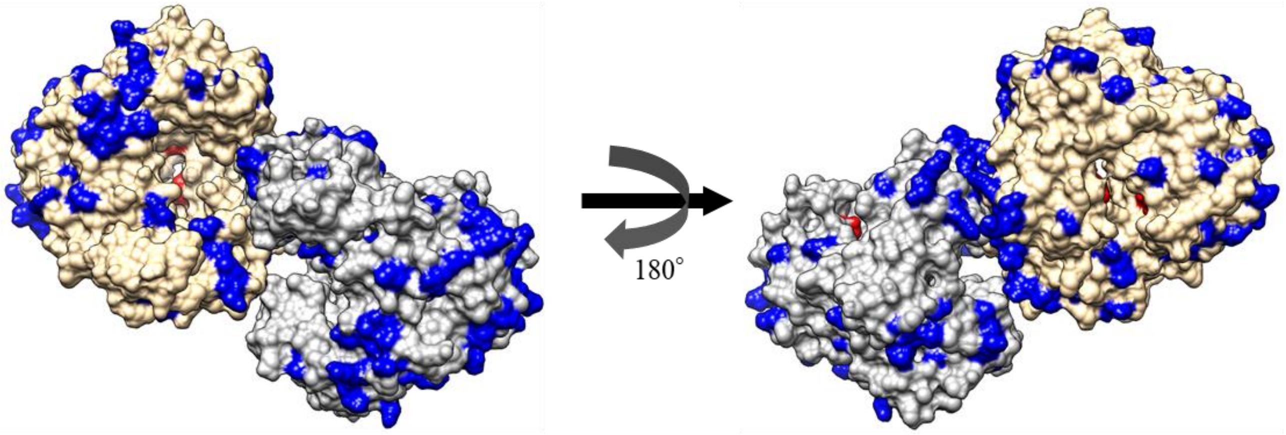

(E) GII.P6

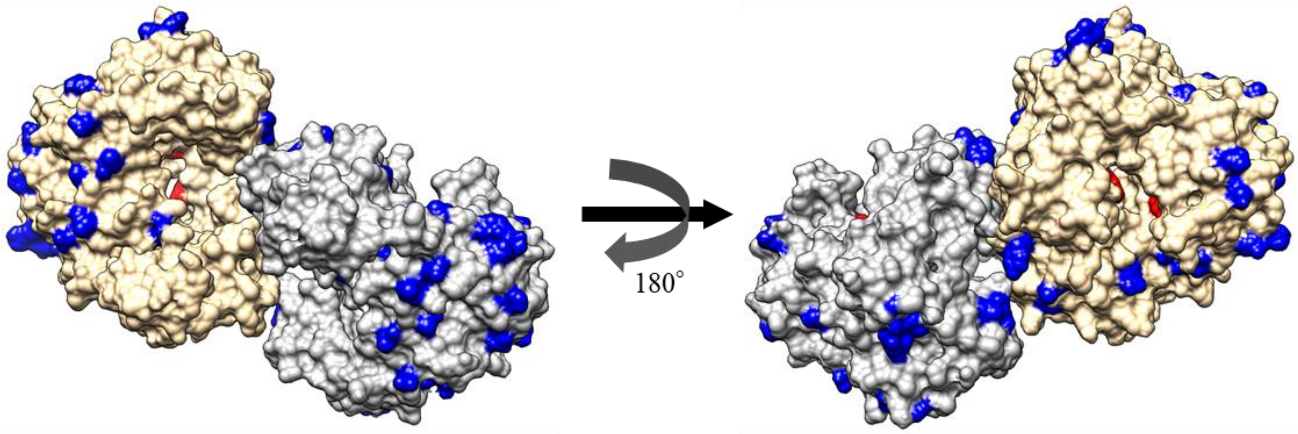

(F) GII.P15

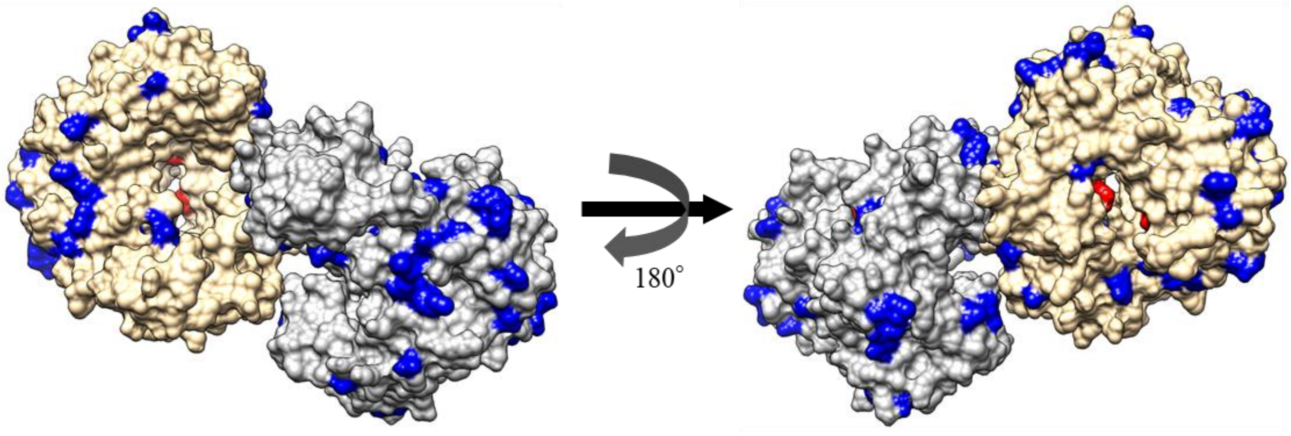

(G) GII.P17

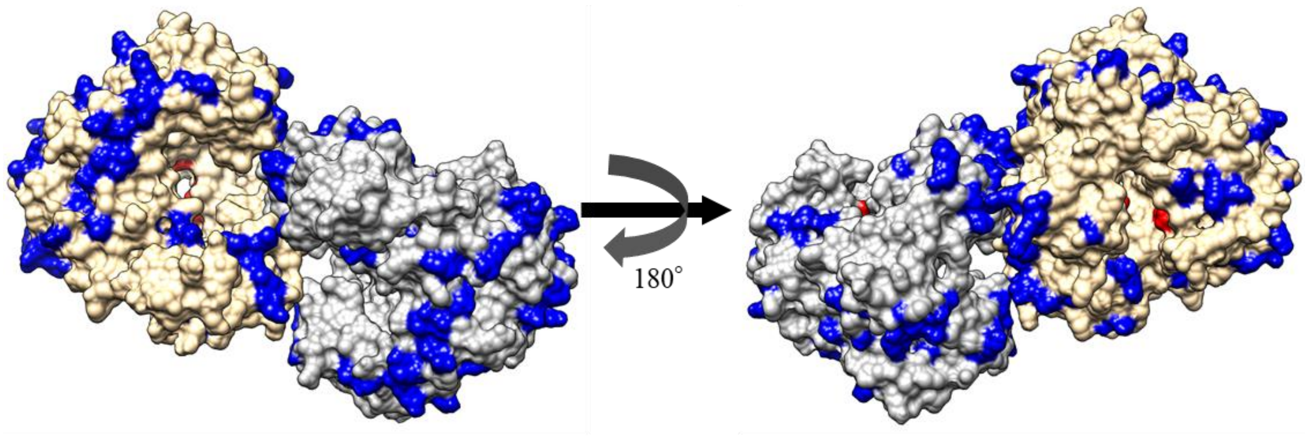

(H) GII.P20

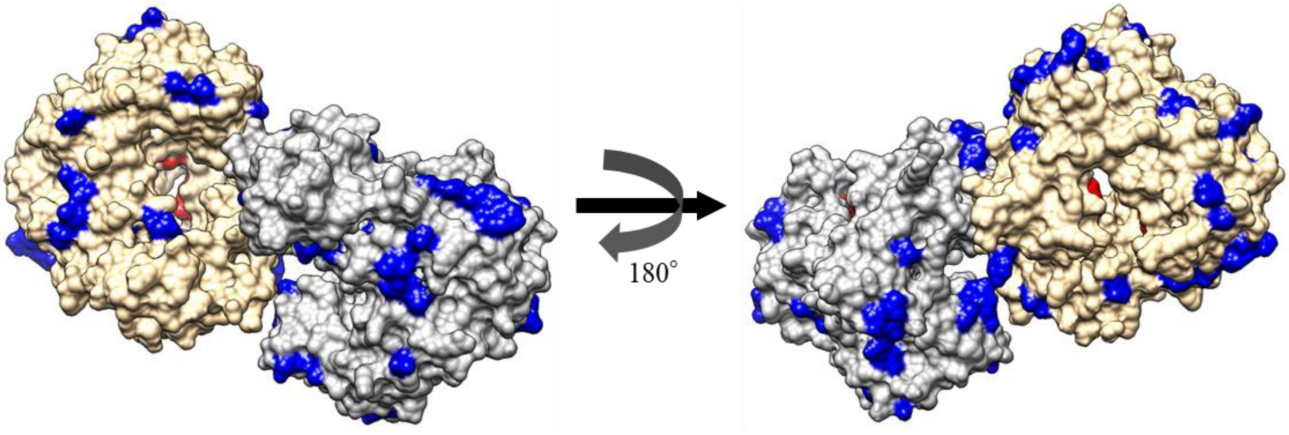

(I) GII.P22

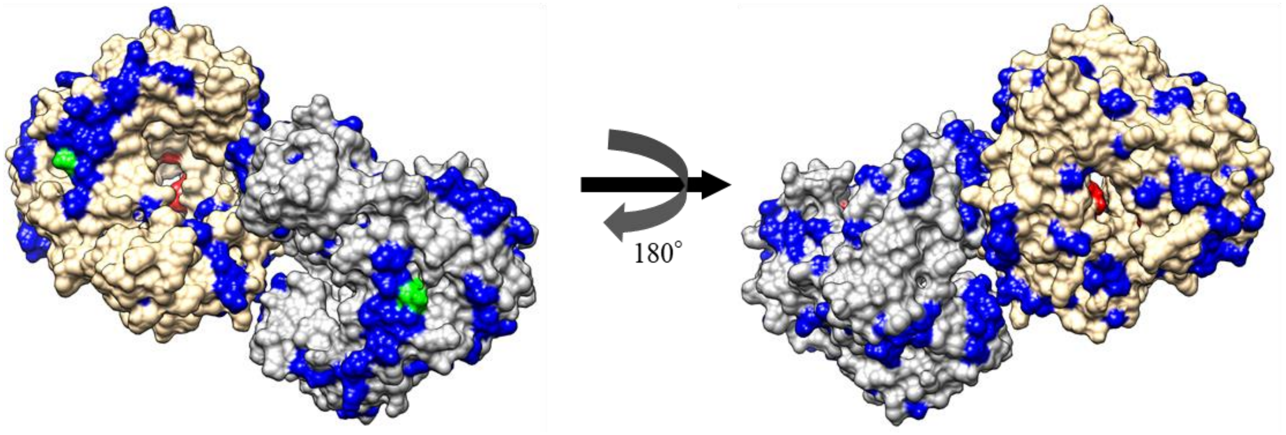

(J) GII.P23

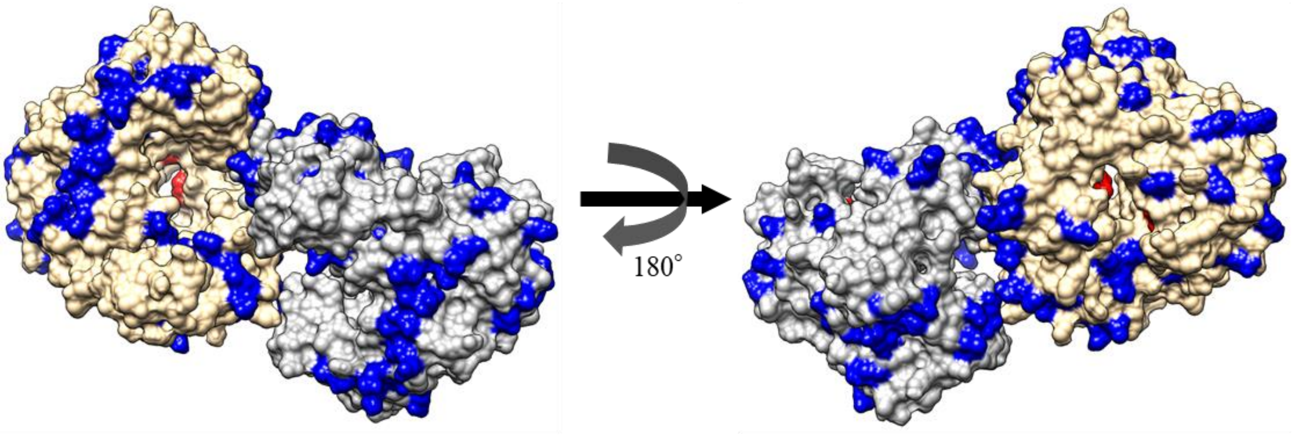

(K) GII.P24

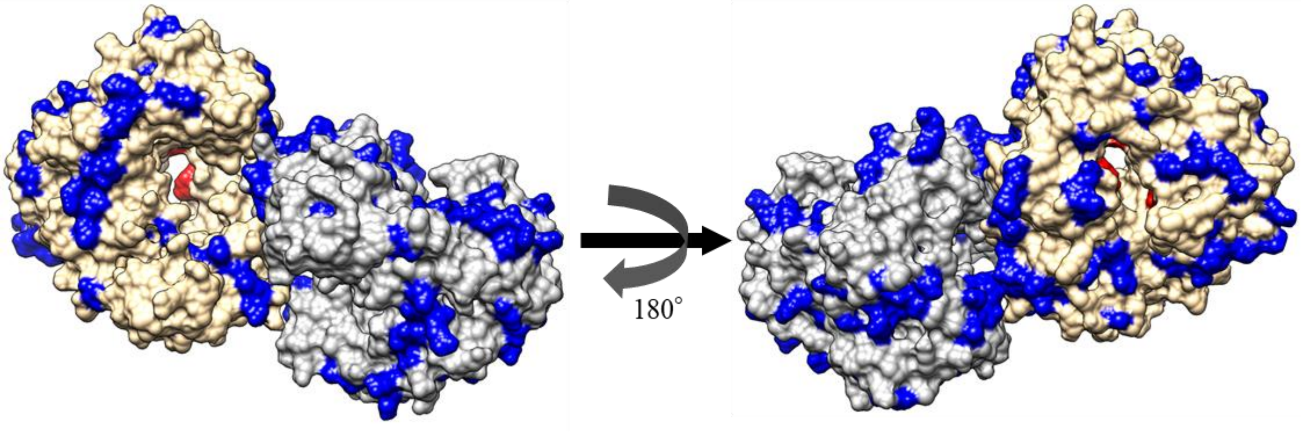

(L) GII.Pc

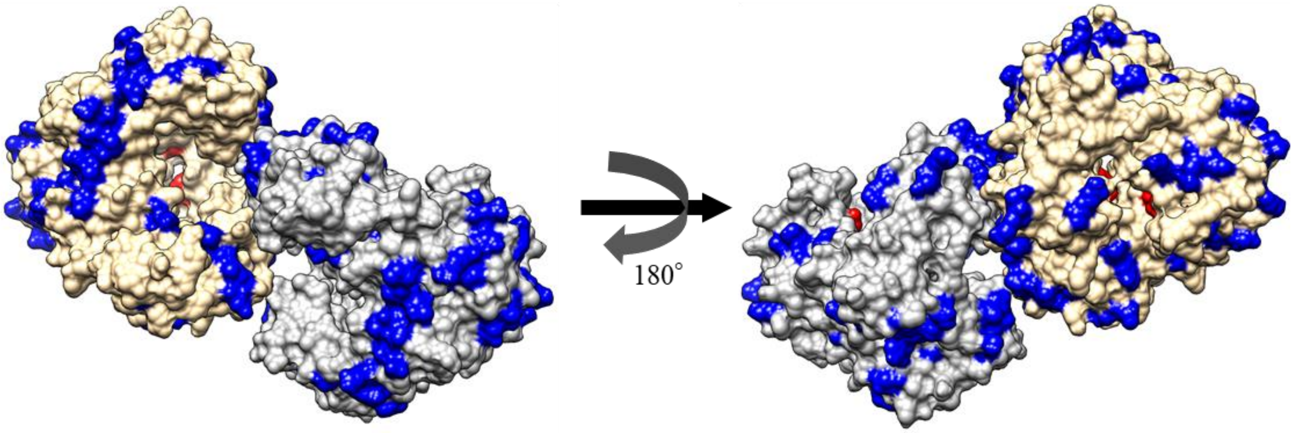

(M) GII.Pf

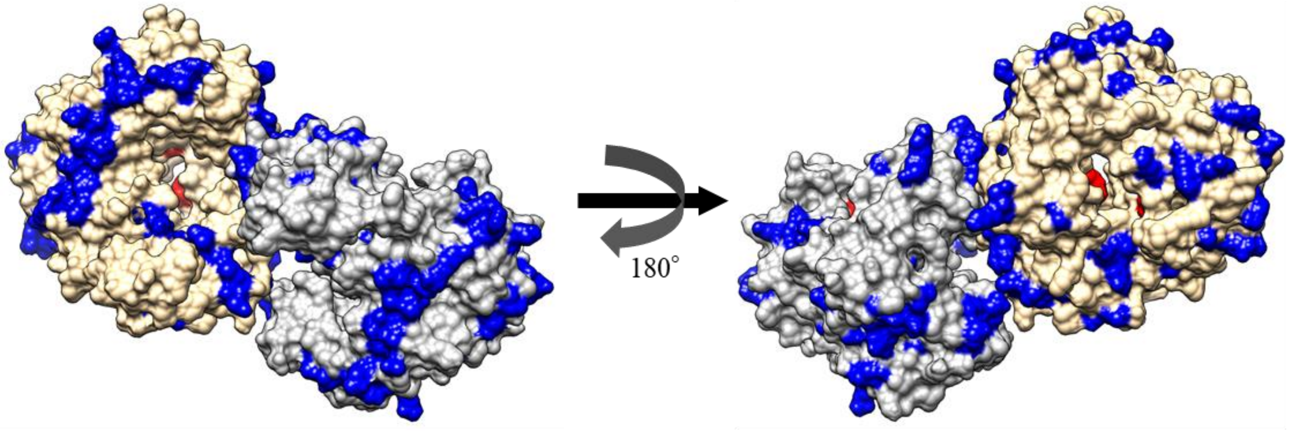

(N) GII.Pg

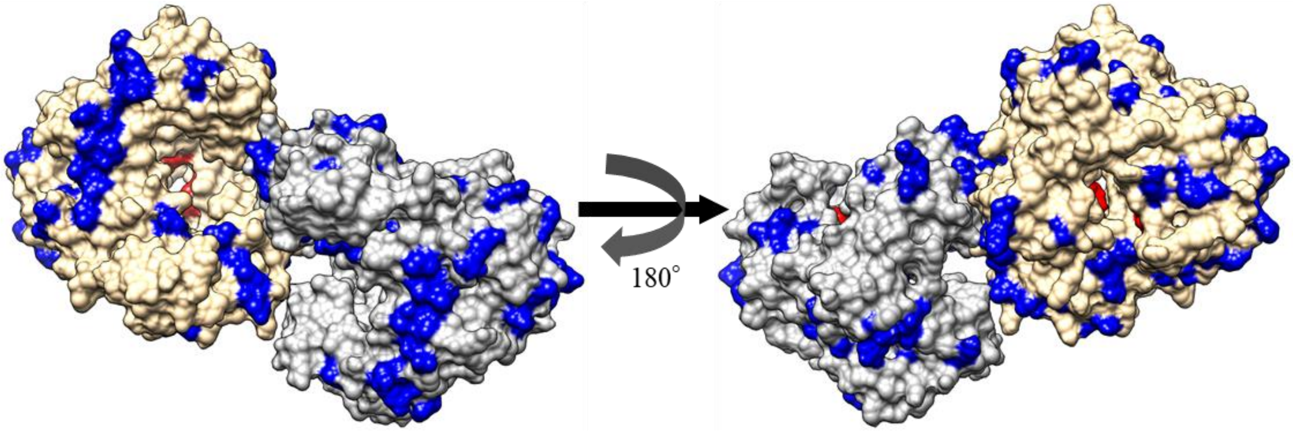

(O) GII.Pj

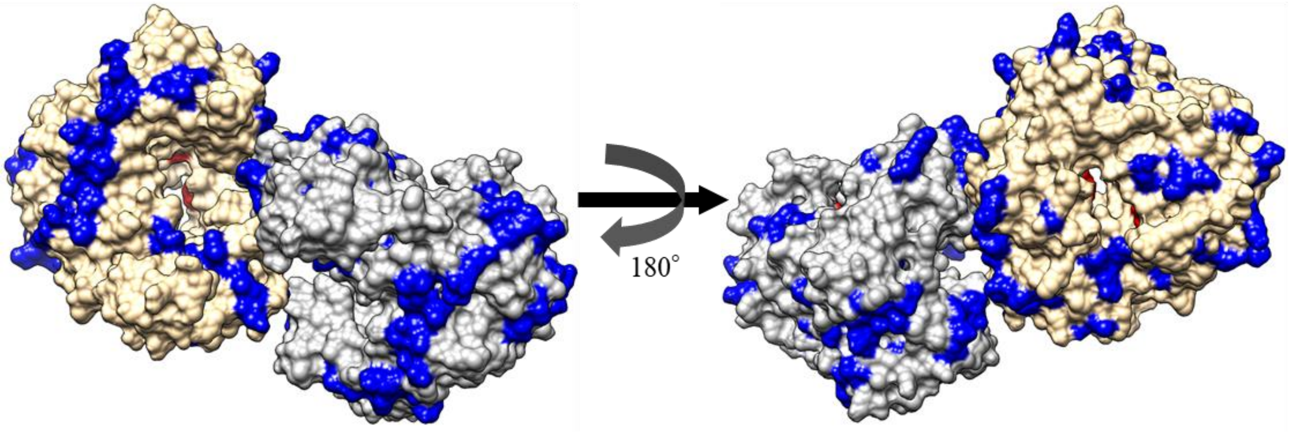

(P) GII.Pm

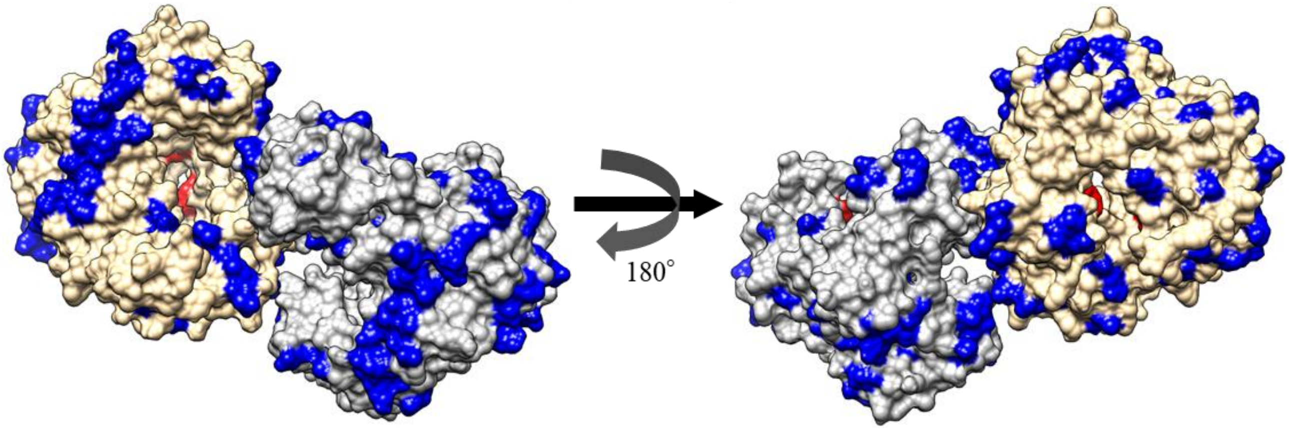

Firugre S2. Structural models for the RdRp protein of each genotype. Three-dimensional RdRp dimer structures for GII.P1 (A), GII.P2 (B), GII.P3 (C), GII.P5 (D), GII.6 (E), GII.P15 (F), GII.P17 (G), GII.P20 (H), GII.P22 (I), GII.P23 (J), GII.P24 (K), GII.Pc (L), GII.Pf (M), GII.Pg (N), GII.Pj (O) and GII.Pm (P) are shown. The chains to composing of the dimer structures are coloured grey (chain A) and Navajo white (chain B). Negative selection sites are coloured green. Amino acid substitutions of the other genotypes compared to a GII.P8 strain are coloured blue. The residues of active site for replication are coloured red.

Supplemental Tables

Table S1. The strains used in this study.

GenBank accession No.	RdRp genotype	Name
AB684675	GII.P1	Hu/GII/JP/1975/GII.P1-GII.4/21-5/Tokyo
**FJ537135**	**GII.P1**	**Hu/GII/US/1974/GII.P1-GII.4/CHDC2094**
FJ537138	GII.P1	Hu/GII/US/1977/GII.P1-GII.4/CHDC4871
JX023286	GII.P1	Hu/GII/US/1974/GII.P1-GII.4/CHDC5191
KC576915	GII.P1	Hu/GII/MYS/1978/GII.P1-GII.4/KL45
U07611	GII.P1	Hu/GII/US/1971/GII.P1-GII.1/Hawaii virus
LC209437	GII.P2	Hu/GII/JP/2004/GII.P2-GII.2/Tochigi-87
LC209440	GII.P2	Hu/GII/JP/2015/GII.P2-GII.2/Saitama-169
LC209457	GII.P2	Hu/GII/JP/2015/GII.P2-GII.2/Miyagi-63
LC209462	GII.P2	Hu/GII/JP/2006/GII.P2-GII.2/Hokkaido-14
LC209463	GII.P2	Hu/GII/JP/2008/GII.P2-GII.2/Hokkaido-15
LC209464	GII.P2	Hu/GII/JP/2004/GII.P2-GII.2/Hokkaido-13
LC209469	GII.P2	Hu/GII/JP/2014/GII.P2-GII.2/Yamaguchi-014
LC209473	GII.P2	Hu/GII/JP/2010/GII.P2-GII.2/Hiroshima-19
AB039782	GII.P3	Hu/GII/JP/1998/GII.P3-GII.3/SaitamaU201
JN176920	GII.P3	Hu/GII/NL/2006/GII.P3/Maastricht021
KJ194500	GII.P3	Hu/GII/NL/1995/GII.P3-GII.3/Amsterdam/1
KJ194504	GII.P3	Hu/GII/NL/1995/GII.P3-GII.3/Amsterdam
AB541266	GII.P4 (Apeldoorn 2007)	Hu/GII/JP/2009/GII.P4-GII.4/Hokkaido4
AB541310	GII.P4 (Apeldoorn 2007)	Hu/GII/JP/2008/GII.P4-GII.4/Niigata1
AB541320	GII.P4 (Apeldoorn 2007)	Hu/GII/JP/2008/GII.P4-GII.4/Osaka1
AB933729	GII.P4 (Apeldoorn 2007)	Hu/GII/JP/2009/GII.P4-GII.4/Fukui5
AB933730	GII.P4 (Apeldoorn 2007)	Hu/GII/JP/2009/GII.P4-GII.4/Ehime1
AB933759	GII.P4 (Apeldoorn 2007)	Hu/GII/JP/2011/GII.P4-GII.4/Nagano26
HM635101	GII.P4 (Apeldoorn 2007)	Hu/GII/KOR/2009/GII.P4-GII.4/Seoul/0921
HM748973	GII.P4 (Apeldoorn 2007)	Hu/GII/AUS/2009/GII.P4-GII.4/NSW892U
HQ009513	GII.P4 (Apeldoorn 2007)	Hu/GII/KOR/2008/GII.P4-GII.4/JB-15
JX439815	GII.P4 (Apeldoorn 2007)	Hu/GII/KOR/2010/GII.P4-GII.4/Seoul1055
JX439818	GII.P4 (Apeldoorn 2007)	Hu/GII/KOR/2010/GII.P4-GII.4/Seoul1367
JX445161	GII.P4 (Apeldoorn 2007)	Hu/GII/CA/2008/GII.P4-GII.4/AlbertaEl210
JX459903	GII.P4 (Apeldoorn 2007)	Hu/GII/AU/2011/GII.P4-GII.4/Jannali/NSW774M
KC409311	GII.P4 (Apeldoorn 2007)	Hu/GII/VNM/2009/GII.P4-GII.4/30199
KC597143	GII.P4 (Apeldoorn 2007)	Hu/GII/USA/2010/GII.P4-GII.4/NIHIC1.2
KF712494	GII.P4 (Apeldoorn 2007)	Hu/GII/USA/2012/GII.P4-GII.4/NIHIC1.7
MF140633	GII.P4 (Apeldoorn 2007)	Hu/GII/NL/2009/GII.P4-GII.4/Rotterdam/E7800007
MF140634	GII.P4 (Apeldoorn 2007)	Hu/GII/NL/2010/GII.P4-GII.4/Rotterdam/E1300272
FJ537137	GII.P4 (Bristol 1993)	Hu/GII/US/1987/GII.P4-GII.4/CHDC4108
X86557	GII.P4 (Bristol 1993)	Hu/GII/UK/1993/GII.P4-GII.4/Lordsdale
AB985442	GII.P4 (Camberwell 1994)	Hu/GII/JPN/1987/GII.P4-GII.4/2-46/Tokyo
JX289821	GII.P4 (Camberwell 1994)	Hu/GII/USA/1987/GII.P4-GII.4/MD120-12
KY424339	GII.P4 (Camberwell 1994)	Hu/GII/US/1988/GII.P4-GII.4/MD04-1A
AB447427	GII.P4 (Den Haag 2006b)	Hu/GII/JP/2006/GII.P4-GII.4/Hokkaido1
AB447428	GII.P4 (Den Haag 2006b)	Hu/GII/JP/2006/GII.P4-GII.4/Hokkaido2
AB447437	GII.P4 (Den Haag 2006b)	Hu/GII/JP/2006/GII.P4-GII.4/Akita2
AB447440	GII.P4 (Den Haag 2006b)	Hu/GII/JP/2006/GII.P4-GII.4/Miyagi2
AB447449	GII.P4 (Den Haag 2006b)	Hu/GII/JP/2006/GII.P4-GII.4/Sakai3
AB447451	GII.P4 (Den Haag 2006b)	Hu/GII/JP/2006/GII.P4-GII.4/Hiroshima1
AB447454	GII.P4 (Den Haag 2006b)	Hu/GII/JP/2006/GII.P4-GII.4/Ehime2
AB541212	GII.P4 (Den Haag 2006b)	Hu/GII/JP/2008/GII.P4-GII.4/Akita1
AB541213	GII.P4 (Den Haag 2006b)	Hu/GII/JP/2008/GII.P4-GII.4/Akita2
AB541214	GII.P4 (Den Haag 2006b)	Hu/GII/JP/2007/GII.P4-GII.4/Akita3
AB541217	GII.P4 (Den Haag 2006b)	Hu/GII/JP/2007/GII.P4-GII.4/Akita5
AB541228	GII.P4 (Den Haag 2006b)	Hu/GII/JP/2007/GII.P4-GII.4/Chiba1
AB541232	GII.P4 (Den Haag 2006b)	Hu/GII/JP/2007/GII.P4-GII.4/Chiba4
AB541234	GII.P4 (Den Haag 2006b)	Hu/GII/JP/2008/GII.P4-GII.4/Chiba5
AB541237	GII.P4 (Den Haag 2006b)	Hu/GII/JP/2008/GII.P4-GII.4/Ehime1

The bold letter strains might be estimated the genotypes in this study.

Table S1 (continued). The strains used in this study.

GenBank accession No.	RdRp genotype	Name
AB541238	GII.P4 (Den Haag 2006b)	Hu/GII/JP/2007/GII.P4-GII.4/Ehime2
AB541240	GII.P4 (Den Haag 2006b)	Hu/GII/JP/2009/GII.P4-GII.4/Ehime3
AB541243	GII.P4 (Den Haag 2006b)	Hu/GII/JP/2009/GII.P4-GII.4/Ehime5
AB541246	GII.P4 (Den Haag 2006b)	Hu/GII/JP/2008/GII.P4-GII.4/Fukui2
AB541247	GII.P4 (Den Haag 2006b)	Hu/GII/JP/2007/GII.P4-GII.4/Fukui4
AB541248	GII.P4 (Den Haag 2006b)	Hu/GII/JP/2008/GII.P4-GII.4/Fukui4
AB541250	GII.P4 (Den Haag 2006b)	Hu/GII/JP/2008/GII.P4-GII.4/Fukui5
AB541252	GII.P4 (Den Haag 2006b)	Hu/GII/JP/2008/GII.P4-GII.4/Hiroshima1
AB541254	GII.P4 (Den Haag 2006b)	Hu/GII/JP/2008/GII.P4-GII.4/Hiroshima2
AB541256	GII.P4 (Den Haag 2006b)	Hu/GII/JP/2008/GII.P4-GII.4/Hiroshima3
AB541258	GII.P4 (Den Haag 2006b)	Hu/GII/JP/2008/GII.P4-GII.4/Hiroshima4
AB541260	GII.P4 (Den Haag 2006b)	Hu/GII/JP/2007/GII.P4-GII.4/Hokkaido1
AB541262	GII.P4 (Den Haag 2006b)	Hu/GII/JP/2007/GII.P4-GII.4/Hokkaido2
AB541263	GII.P4 (Den Haag 2006b)	Hu/GII/JP/2008/GII.P4-GII.4/Hokkaido2
AB541264	GII.P4 (Den Haag 2006b)	Hu/GII/JP/2008/GII.P4-GII.4/Hokkaido3
AB541276	GII.P4 (Den Haag 2006b)	Hu/GII/JP/2008/GII.P4-GII.4/Iwate5
AB541279	GII.P4 (Den Haag 2006b)	Hu/GII/JP/2007/GII.P4-GII.4/Kumamoto3
AB541289	GII.P4 (Den Haag 2006b)	Hu/GII/JP/2008/GII.P4-GII.4/Miyazaki1
AB541290	GII.P4 (Den Haag 2006b)	Hu/GII/JP/2007/GII.P4-GII.4/Miyazaki2
AB541304	GII.P4 (Den Haag 2006b)	Hu/GII/JP/2009/GII.P4-GII.4/Nagano2
AB541311	GII.P4 (Den Haag 2006b)	Hu/GII/JP/2007/GII.P4-GII.4/Niigata2
AB541314	GII.P4 (Den Haag 2006b)	Hu/GII/JP/2009/GII.P4-GII.4/Niigata3
AB541326	GII.P4 (Den Haag 2006b)	Hu/GII/JP/2008/GII.P4-GII.4/Osaka4
AB541333	GII.P4 (Den Haag 2006b)	Hu/GII/JP/2008/GII.P4-GII.4/Saga2
AB541334	GII.P4 (Den Haag 2006b)	Hu/GII/JP/2009/GII.P4-GII.4/Saga3
AB541343	GII.P4 (Den Haag 2006b)	Hu/GII/JP/2008/GII.P4-GII.4/Sakai3
AB541355	GII.P4 (Den Haag 2006b)	Hu/GII/JP/2007/GII.P4-GII.4/Toyama2
AB933652	GII.P4 (Den Haag 2006b)	Hu/GII/JP/2009/GII.P4-GII.4/Iwate2
AB933655	GII.P4 (Den Haag 2006b)	Hu/GII/JP/2009/GII.P4-GII.4/Shimane2
AB933663	GII.P4 (Den Haag 2006b)	Hu/GII/JP/2010/GII.P4-GII.4/Chiba5
AB933666	GII.P4 (Den Haag 2006b)	Hu/GII/JP/2010/GII.P4-GII.4/Osaka2
AB933675	GII.P4 (Den Haag 2006b)	Hu/GII/JP/2010/GII.P4-GII.4/Hokkaido4
AB933687	GII.P4 (Den Haag 2006b)	Hu/GII/JP/2010/GII.P4-GII.4/Shimane4
AB933690	GII.P4 (Den Haag 2006b)	Hu/GII/JP/2010/GII.P4-GII.4/Miyazaki4
AB933708	GII.P4 (Den Haag 2006b)	Hu/GII/JP/2011/GII.P4-GII.4/Hiroshimacity3
AB933714	GII.P4 (Den Haag 2006b)	Hu/GII/JP/2011/GII.P4-GII.4/Iwate2
AB933716	GII.P4 (Den Haag 2006b)	Hu/GII/JP/2011/GII.P4-GII.4/Iwate5
AB933724	GII.P4 (Den Haag 2006b)	Hu/GII/JP/2011/GII.P4-GII.4/Aichi3
EF684915	GII.P4 (Den Haag 2006b)	Hu/GII/AUS/2006/GII.P4-GII.4/Shellharbour/NSW696T
FJ514242	GII.P4 (Den Haag 2006b)	Hu/GII/KR/2008/GII.P4-GII.4/CUK-3
GQ845024	GII.P4 (Den Haag 2006b)	Hu/GII/AUS/2007/GII.P4-GII.4/Rathmines/NSW287R
GQ845322	GII.P4 (Den Haag 2006b)	Hu/GII/AU/2007/GII.P4-GII.4/VIC3863
GQ845366	GII.P4 (Den Haag 2006b)	Hu/GII/AUS/2008/GII.P4-GII.4/Westmead/NSW3639
GU325839	GII.P4 (Den Haag 2006b)	Hu/GII/US/2009/GII.P4-GII.4/HS194
GU991353	GII.P4 (Den Haag 2006b)	Hu/GII/CHN/2008/GII.P4-GII.4/Shanghai/SH2
HM748971	GII.P4 (Den Haag 2006b)	Hu/GII/AUS/2009/GII.P4-GII.4/Beecroft/NSW305P
JN400600	GII.P4 (Den Haag 2006b)	Hu/GII/TW/2006/GII.P4-GII.4/CGMH02
JN400602	GII.P4 (Den Haag 2006b)	Hu/GII/TW/2006/GII.P4-GII.4/CGMH04
JN400613	GII.P4 (Den Haag 2006b)	Hu/GII/TW/2007/GII.P4-GII.4/CGMH15
JN400616	GII.P4 (Den Haag 2006b)	Hu/GII/TW/2008/GII.P4-GII.4/CGMH18
JN400617	GII.P4 (Den Haag 2006b)	Hu/GII/TW/2009/GII.P4-GII.4/CGMH19
JN400620	GII.P4 (Den Haag 2006b)	Hu/GII/TW/2010/GII.P4-GII.4/CGMH22
JQ613572	GII.P4 (Den Haag 2006b)	Hu/GII/AU/2010/GII.P4-GII.4/StVincents/NSW217
JQ911595	GII.P4 (Den Haag 2006b)	Hu/GII/VNM/2009/GII.P4-GII.4/10002
JQ911597	GII.P4 (Den Haag 2006b)	Hu/GII/VNM/2009/GII.P4-GII.4/10012
JX445160	GII.P4 (Den Haag 2006b)	Hu/GII/CA/2008/GII.P4-GII.4/AlbertaEI102
JX445163	GII.P4 (Den Haag 2006b)	Hu/GII/CA/2009/GII.P4-GII.4/AlbertaEI109
JX459900	GII.P4 (Den Haag 2006b)	Hu/GII/AU/2011/GII.P4-GII.4/Randwick/NSW882J
JX459905	GII.P4 (Den Haag 2006b)	Hu/GII/AU/2011/GII.P4-GII.4/Randwick/NSW938K

Table S1 (continued). The strains used in this study.

GenBank accession No.	RdRp genotype	Name
JX459906	GII.P4 (Den Haag 2006b)	Hu/GII/AU/2011/GII.P4-GII.4/Miranda/NSW850K
JX989073	GII.P4 (Den Haag 2006b)	Hu/GII/CHN/2010/GII.P4-GII.4/GZ2010-L26/Guangzhou
KC175348	GII.P4 (Den Haag 2006b)	Hu/GII/VNM/2009/GII.P4-GII.4/10078
KC175353	GII.P4 (Den Haag 2006b)	Hu/GII/VNM/2009/GII.P4-GII.4/10129
KC175374	GII.P4 (Den Haag 2006b)	Hu/GII/VNM/2009/GII.P4-GII.4/10223
KC175377	GII.P4 (Den Haag 2006b)	Hu/GII/VNM/2009/GII.P4-GII.4/10238
KC175378	GII.P4 (Den Haag 2006b)	Hu/GII/VNM/2009/GII.P4-GII.4/10247
KC175379	GII.P4 (Den Haag 2006b)	Hu/GII/VNM/2009/GII.P4-GII.4/10255
KC175397	GII.P4 (Den Haag 2006b)	Hu/GII/VNM/2009/GII.P4-GII.4/20066
KC175402	GII.P4 (Den Haag 2006b)	Hu/GII/VNM/2009/GII.P4-GII.4/20094
KC175403	GII.P4 (Den Haag 2006b)	Hu/GII/VNM/2009/GII.P4-GII.4/20118
KC409256	GII.P4 (Den Haag 2006b)	Hu/GII/VNM/2009/GII.P4-GII.4/20156
KC409263	GII.P4 (Den Haag 2006b)	Hu/GII/VNM/2009/GII.P4-GII.4/20173
KC409291	GII.P4 (Den Haag 2006b)	Hu/GII/VNM/2009/GII.P4-GII.4/20350
KC409293	GII.P4 (Den Haag 2006b)	Hu/GII/VNM/2010/GII.P4-GII.4/20365
KC409314	GII.P4 (Den Haag 2006b)	Hu/GII/VNM/2009/GII.P4-GII.4/30207
KC517368	GII.P4 (Den Haag 2006b)	Hu/GII/TW/2012/GII.P4-GII.4/New Taipei/CGMH58
KC517369	GII.P4 (Den Haag 2006b)	Hu/GII/TW/2012/GII.P4-GII.4/Taoyuan/CGMH59
KC576909	GII.P4 (Den Haag 2006b)	Hu/GII/USA/2011/GII.P4-GII.4/NIHIC4.2
KC810027	GII.P4 (Den Haag 2006b)	Hu/GII/GBR/2010/GII.P4-GII.4/clone PxA040710
KC894942	GII.P4 (Den Haag 2006b)	Hu/GII/CHN/2011/GII.P4-GII.4/Guanzhou/GZ2010-L88
KC960614	GII.P4 (Den Haag 2006b)	Hu/GII/VNM/2009/GII.P4-GII.4/30116
KF429776	GII.P4 (Den Haag 2006b)	Hu/GII/USA/2012/GII.P4-GII.4/NIHIC17.1
KM198562	GII.P4 (Den Haag 2006b)	Hu/GII/VNM/2011/GII.P4-GII.4/C2H-55
KM198568	GII.P4 (Den Haag 2006b)	Hu/GII/VNM/2011/GII.P4-GII.4/C2H-62
KM245074	GII.P4 (Den Haag 2006b)	Hu/GII/VNM/2009/GII.P4-GII.4/09-N-1
KT152148	GII.P4 (Den Haag 2006b)	Hu/GII/USA/2006/GII.P4-GII.4/Minerva
KU311162	GII.P4 (Den Haag 2006b)	Hu/GII/CA/2012/GII.P4-GII.4/AlbertaSP1
KX514351	GII.P4 (Den Haag 2006b)	Hu/GII/GE/2014/GII.P4-GII.4/2.6RdRpVP1
KX514352	GII.P4 (Den Haag 2006b)	Hu/GII/GE/2014/GII.P4-GII.4/2.5 RdRpVP1
KX514356	GII.P4 (Den Haag 2006b)	Hu/GII/GE/2014/GII.P4-GII.4/4.6 RdRpVP1
KX514358	GII.P4 (Den Haag 2006b)	Hu/GII/GE/2015/GII.P4-GII.4/11.8 RdRpVP1
KX514359	GII.P4 (Den Haag 2006b)	Hu/GII/GE/2015/GII.P4-GII.4/11.7 RdRpVP1
KX514361	GII.P4 (Den Haag 2006b)	Hu/GII/GE/2015/GII.P4-GII.4/11.4 RdRpVP1
KX514362	GII.P4 (Den Haag 2006b)	Hu/GII/GE/2015/GII.P4-GII.4/11.1 RdRpVP1
KX514369	GII.P4 (Den Haag 2006b)	Hu/GII/GE/2015/GII.P4-GII.4/12.2 RdRpVP1
MF140652	GII.P4 (Den Haag 2006b)	Hu/GII/NL/2008/GII.P4-GII.4/Rotterdam/E7800015
MG049692	GII.P4 (Den Haag 2006b)	Hu/GII/TW/2009/GII.P4-GII.4/YJB1
AY587983	GII.P4 (Farmington Hills 2002)	Hu/GII/UK/2002/GII.P4-GII.4/Oxford/B4S2
DQ415279	GII.P4 (Farmington Hills 2002)	Hu/GII/Ire/2002/GII.P4-GII.4/Carlow
DQ658413	GII.P4 (Farmington Hills 2002)	Hu/GII/US/2004/GII.P4-GII.4/MD-2004
EF202567	GII.P4 (Farmington Hills 2002)	Hu/GII/CAN/2002/GII.P4-GII.4/Toronto/SK
JQ798158	GII.P4 (Farmington Hills 2002)	Hu/GII/USA/2004/GII.P4-GII.4/5M
JX126912	GII.P4 (Farmington Hills 2002)	Hu/GII/USA/2012/GII.P4-GII.4/Ohio/7
JX445152	GII.P4 (Farmington Hills 2002)	Hu/GII/CA/2004/GII.P4-GII.4/AlbertaEI131
EF202568	GII.P4 (Hunter 2004)	Hu/GII/CAN/2005/GII.P4-GII.4/Toronto/SK
EU921344	GII.P4 (Hunter 2004)	Hu/GII/India/2006/GII.P4-GII.4/Pune/PC15
HM802541	GII.P4 (Hunter 2004)	Hu/GII/HKG/2004/GII.P4-GII.4/CU041213
HM802544	GII.P4 (Hunter 2004)	Hu/GII/HKG/2005/GII.P4-GII.4/CU051146
HM802545	GII.P4 (Hunter 2004)	Hu/GII/HKG/2005/GII.P4-GII.4/CU050431
JX445153	GII.P4 (Hunter 2004)	Hu/GII/CA/2006/GII.P4-GII.4/AlbertaEI142
AB933731	GII.P4 (New Orleans 2009)	Hu/GII/JP/2009/GII.P4-GII.4/Hokkaido1
AB933732	GII.P4 (New Orleans 2009)	Hu/GII/JP/2009/GII.P4-GII.4/Hokkaido2
AB933740	GII.P4 (New Orleans 2009)	Hu/GII/JP/2009/GII.P4-GII.4/Chiba2
AB933742	GII.P4 (New Orleans 2009)	Hu/GII/JP/2009/GII.P4-GII.4/Nagano2
AB933746	GII.P4 (New Orleans 2009)	Hu/GII/JP/2009/GII.P4-GII.4/Osaka1
AB933747	GII.P4 (New Orleans 2009)	Hu/GII/JP/2009/GII.P4-GII.4/Osaka5
AB933755	GII.P4 (New Orleans 2009)	Hu/GII/JP/2009/GII.P4-GII.4/Saga2
AB933758	GII.P4 (New Orleans 2009)	Hu/GII/JP/2011/GII.P4-GII.4/Chiba6

Table S1 (continued). The strains used in this study.

GenBank accession No.	RdRp genotype	Name
AB933761	GII.P4 (New Orleans 2009)	Hu/GII/JP/2011/GII.P4-GII.4/Osaka4
AB933763	GII.P4 (New Orleans 2009)	Hu/GII/JP/2011/GII.P4-GII.4/Osaka2
AB933764	GII.P4 (New Orleans 2009)	Hu/GII/JP/2011/GII.P4-GII.4/Osaka1
AB933767	GII.P4 (New Orleans 2009)	Hu/GII/JP/2011/GII.P4-GII.4/Hokkaido3
GQ845367	GII.P4 (New Orleans 2009)	Hu/GII/AU/2008/GII.P4-GII.4/Orange/NSW001P
GU445325	GII.P4 (New Orleans 2009)	Hu/GII/USA/2009/GII.P4-GII.4/New Orleans1805
HF952120	GII.P4 (New Orleans 2009)	Hu/GII/UK/2011/GII.P4-GII.4/C00007876
HF952122	GII.P4 (New Orleans 2009)	Hu/GII/UK/2011/GII.P4-GII.4/C00007880
HF952123	GII.P4 (New Orleans 2009)	Hu/GII/UK/2011/GII.P4-GII.4/C00007881
HF952134	GII.P4 (New Orleans 2009)	Hu/GII/UK/2011/GII.P4-GII.4/C00007941
HM748972	GII.P4 (New Orleans 2009)	Hu/GII/AUS/2009/GII.P4-GII.4/Teralba/NSW881Z
JN400618	GII.P4 (New Orleans 2009)	Hu/GII/TW/2009/GII.P4-GII.4/CGMH20
JN400622	GII.P4 (New Orleans 2009)	Hu/GII/TW/2010/GII.P4-GII.4/CGMH24
JN400624	GII.P4 (New Orleans 2009)	Hu/GII/TW/2010/GII.P4-GII.4/CGMH26
JQ613552	GII.P4 (New Orleans 2009)	Hu/GII/AU/2010/GII.P4-GII.4/NSW123B
JQ613570	GII.P4 (New Orleans 2009)	Hu/GII/AU/2009/GII.P4-GII.4/Rockdale/NSW006D
JQ613571	GII.P4 (New Orleans 2009)	Hu/GII/AU/2010/GII.P4-GII.4/Miranda/NSW817L
JX439817	GII.P4 (New Orleans 2009)	Hu/GII/KOR/2010/GII.P4-GII.4/Seoul1282
JX439819	GII.P4 (New Orleans 2009)	Hu/GII/KOR/2011/GII.P4-GII.4/Seoul1488
JX445166	GII.P4 (New Orleans 2009)	Hu/GII/CA/2010/GII.P4-GII.4/AlbertaEI204
JX445168	GII.P4 (New Orleans 2009)	Hu/GII/CA/2011/GII.P4-GII.4/AlbertaEI388
JX448566	GII.P4 (New Orleans 2009)	Hu/GII/KOR/2010/GII.P4-GII.4/Seoul/1071
JX459904	GII.P4 (New Orleans 2009)	Hu/GII/AU/2011/GII.P4-GII.4/Doonside/NSW536I
JX846928	GII.P4 (New Orleans 2009)	Hu/GII/USA/2011/GII.P4-GII.4/NIHIC9
JX989074	GII.P4 (New Orleans 2009)	Hu/GII/CHN/2011/GII.P4-GII.4/GZ2010-L87/Guangzhou
KC409240	GII.P4 (New Orleans 2009)	Hu/GII/VNM/2010/GII.P4-GII.4/10370
KC409241	GII.P4 (New Orleans 2009)	Hu/GII/VNM/2010/GII.P4-GII.4/10405
KC409242	GII.P4 (New Orleans 2009)	Hu/GII/VNM/2010/GII.P4-GII.4/10406
KC409301	GII.P4 (New Orleans 2009)	Hu/GII/VNM/2010/GII.P4-GII.4/20469
KC409302	GII.P4 (New Orleans 2009)	Hu/GII/VNM/2010/GII.P4-GII.4/20477
KC463910	GII.P4 (New Orleans 2009)	Hu/GII/USA/2012/GII.P4-GII.4/Ohio/684
KC577174	GII.P4 (New Orleans 2009)	Hu/GII/CHN/2011/GII.P4-GII.4/Jiangsu1
KC962462	GII.P4 (New Orleans 2009)	Hu/GII/ZAF/2011/GII.P4-GII.4/Empangeni/8491
KF429766	GII.P4 (New Orleans 2009)	Hu/GII/USA/2011/GII.P4-GII.4/NIHIC13
KF429777	GII.P4 (New Orleans 2009)	Hu/GII/USA/2012/GII.P4-GII.4/NIHIC27.1
KF429788	GII.P4 (New Orleans 2009)	Hu/GII/USA/2012/GII.P4-GII.4/NIHIC20
KF509947	GII.P4 (New Orleans 2009)	Hu/GII/CA/2011/GII.P4-GII.4/AlbertaEI337
KJ196285	GII.P4 (New Orleans 2009)	Hu/GII/TW/2012/GII.P4-GII.4/Taipei/108
KJ407073	GII.P4 (New Orleans 2009)	Hu/GII/USA/2012/GII.P4-GII.4/HS292
KJ407075	GII.P4 (New Orleans 2009)	Hu/GII/USA/2012/GII.P4-GII.4/HS288
KJ685402	GII.P4 (New Orleans 2009)	Hu/GII/BGD/2012/GII.P4-GII.4/BG1C0434
KJ685405	GII.P4 (New Orleans 2009)	Hu/GII/BGD/2011/GII.P4-GII.4/BG1C0282
KJ685408	GII.P4 (New Orleans 2009)	Hu/GII/BGD/2011/GII.P4-GII.4/BG1C0066
KJ685413	GII.P4 (New Orleans 2009)	Hu/GII/BGD/2011/GII.P4-GII.4/BG1C0317
KJ685414	GII.P4 (New Orleans 2009)	Hu/GII/BGD/2010/GII.P4-GII.4/BG1C0004
KJ710245	GII.P4 (New Orleans 2009)	Hu/GII/ZAF/2011/GII.P4-GII.4/CapeTown/6745
KM198555	GII.P4 (New Orleans 2009)	Hu/GII/VNM/2010/GII.P4-GII.4/C2035
KP784691	GII.P4 (New Orleans 2009)	Hu/GII/ZAF/2009/GII.P4-GII.4/Johannesburg/4175
KP784692	GII.P4 (New Orleans 2009)	Hu/GII/ZAF/2011/GII.P4-GII.4/Johannesburg/7028
KP784693	GII.P4 (New Orleans 2009)	Hu/GII/ZAF/2011/GII.P4-GII.4/Empangeni/8501
KP784694	GII.P4 (New Orleans 2009)	Hu/GII/ZAF/2012/GII.P4-GII.4/Empangeni/8604
KP784695	GII.P4 (New Orleans 2009)	Hu/GII/ZAF/2012/GII.P4-GII.4/Empangeni/9693
KP784698	GII.P4 (New Orleans 2009)	Hu/GII/ZAF/2012/GII.P4-GII.4/Johannesburg/BW
KY905331	GII.P4 (New Orleans 2009)	Hu/GII/AU/2016/GII.P4-GII.4/NSW789Z
KY947546	GII.P4 (New Orleans 2009)	Hu/GII/USA/2015/GII.P4-GII.4/Titusville7426
MF140641	GII.P4 (New Orleans 2009)	Hu/GII/NL/2013/GII.P4-GII.4/E7800009
MF140643	GII.P4 (New Orleans 2009)	Hu/GII/NL/2013/GII.P4-GII.4/E1300307
MF140649	GII.P4 (New Orleans 2009)	Hu/GII/NL/2010/GII.P4-GII.4/E7800012
MF140669	GII.P4 (New Orleans 2009)	Hu/GII/NL/2012/GII.P4-GII.4/E1300296

Table S1 (continued). The strains used in this study.

GenBank accession No.	RdRp genotype	Name
MF140693	GII.P4 (New Orleans 2009)	Hu/GII/NL/2011/GII.P4-GII.4/E7800020
MF140694	GII.P4 (New Orleans 2009)	Hu/GII/NL/2013/GII.P4-GII.4/E1300280
MF140697	GII.P4 (New Orleans 2009)	Hu/GII/NL/2014/GII.P4-GII.4/E1300283
MG002632	GII.P4 (New Orleans 2009)	Hu/GII/AU/2017/GII.P4-GII.4/BNE3
AY741811	GII.P4 (US95_96)	Hu/GII/GE/1997/GII.P4-GII.4/Dresden174
KJ407076	GII.P4 (US95_96)	Hu/GII/USA/2001/GII.P4-GII.4/HS66
AB447432	GII.P4 (Yerseke 2006a)	Hu/GII/JP/2006/GII.P4-GII.4/Aomori1
AB447458	GII.P4 (Yerseke 2006a)	Hu/GII/JP/2006/GII.P4-GII.4/Saga5
AB541267	GII.P4 (Yerseke 2006a)	Hu/GII/JP/2008/GII.P4-GII.4/Hokkaido5
KC631815	GII.P4 (Yerseke 2006a)	Hu/GII/USA/2006/GII.P4-GII.4/MI002
KM245069	GII.P4 (Yerseke 2006a)	Hu/GII/TW/2006/GII.P4-GII.4/06-AM-11
**AB684704**	**GII.P4**	**Hu/GII/JP/1980/GII.P4-GII.4/52-2/Tokyo**
**AB684705**	**GII.P4**	**Hu/GII/JP/1980/GII.P4-GII.4/53-1/Tokyo**
**AB985437**	**GII.P4**	**Hu/GII/JP/1987/GII.P4-GII.4/2-37/Tokyo**
EF187497	GII.P4	Hu/GII/NZL/2006/GII.P4-GII.4/NZ327
FJ537136	GII.P4	Hu/GII/US/1988/GII.P4-GII.4/CHDC3967
**GQ845368**	**GII.P4**	**Hu/GII/AUS/2007/GII.P4-GII.4/NSW505G**
HM635100	GII.P4	Hu/GII/KOR/2009/GII.P4-GII.4/Seoul/0654
JN176921	GII.P4	Hu/GII/NL/2002/GII.P4/WeertE022
JN176923	GII.P4	Hu/GII/NL/2006/GII.P4/Temeuzen070
JN400599	GII.P4	Hu/GII/TW/2006/GII.P4-GII.4/CGMH01
JX445154	GII.P4	Hu/GII/CA/2006/GII.P4-GII.4/AlbertaEI190
**KC597139**	**GII.P4**	**Hu/GII/GUF/1978/GII.P4-GII.17/C142**
KC810031	GII.P4	Hu/GII/UK/2010/GII.P4-GII.4/PxE230710
KC962453	GII.P4	Hu/GII/ZAF/2010/GII.P4-GII.4/Bushbuckridge/5928
KF429760	GII.P4	Hu/GII/USA/2012/GII.P4-GII.4/NIHIC28.4
KY947547	GII.P4	Hu/GII/USA/2014/GII.P4-GII.4/Ellsworth 7118
MF140658	GII.P4	Hu/GII/NL/2009/GII.P4-GII.4/E1300265
MF140659	GII.P4	Hu/GII/NL/2009/GII.P4-GII.4/E1300266
MF140662	GII.P4	Hu/GII/NL/2010/GII.P4-GII.4/E1300269
MF140664	GII.P4	Hu/GII/NL/2010/GII.P4-GII.4/E7800014
MF140666	GII.P4	Hu/GII/NL/2011/GII.P4-GII.4/E1300289
MF140667	GII.P4	Hu/GII/NL/2011/GII.P4-GII.4/E1300290
KJ196288	GII.P5	Hu/GII/JP/2002/GII.P5-GII.5/Saitama/T52
AB039778	GII.P6	Hu/GII/JP/1997/GII.P6-GII.6/Saitama U16
HQ169542	GII.P6	Hu/GII/USA/2005/GII.P6-GII.6/186
JX989075	GII.P6	Hu/GII/CHN/2011/GII.P6-GII.6/GZ2010-L96
**KC576910**	**GII.P6**	**Hu/GII/SEN/1976/GII.P6-GII.6/S9c**
**KC597146**	**GII.P6**	**Hu/GII/USA/1975/GII.P6-GII.6/CHDC2685**
KY424345	GII.P6	Hu/GII/US/1971/GII.P6-GII.6/HenrytonH1
AB039777	GII.P7	Hu/GII/JP/1997/GII.P7-GII.6/Saitama U4
EF670650	GII.P7	Hu/GII/CHN/2006/GII.P7-GII.14/Shanxi/50106
GQ849131	GII.P7	Hu/GII/TH/2002/GII.P7/Mc17
GU017900	GII.P7	Hu/GII/JP/2007/GII.P7-GII.14/8434/Maizuru
GU017905	GII.P7	Hu/GII/JP/2008/GII.P7-GII.14/8560/Maizuru
GU930737	GII.P7	Hu/GII/USA/1997/GII.P7-GII.6/E99-13646
JX846927	GII.P7	Hu/GII/USA/1984/GII.P7-GII.6/CHDC4073
KJ196278	GII.P7	Hu/GII/JP/2007/GII.P7-GII.14/Sendai/YG99
KJ196295	GII.P7	Hu/GII/JP/2010/GII.P7-GII.7/TAKAsanKimchi
KJ407072	GII.P7	Hu/GII/USA/2010/GII.P7-GII.6/HS245
KM198498	GII.P7	Hu/GII/VNM/2009/GII.P7-GII.6/20088
KM198519	GII.P7	Hu/GII/VNM/2009/GII.P7-GII.6/30082
KM198530	GII.P7	Hu/GII/VNM/2010/GII.P7-GII.6/20486
KM198531	GII.P7	Hu/GII/VNM/2009/GII.P7-GII.6/30116
KM267740	GII.P7	Hu/GII/TW/2013/GII.P7-GII.6/Kaohsiung/13-BP-2
KM267741	GII.P7	Hu/GII/TW/2013/GII.P7-GII.6/Yilan/13-BT-4
KM267743	GII.P7	Hu/GII/TW/2013/GII.P7-GII.6/Kaohsiung/13-BV-1
KM461690	GII.P7	Hu/GII/TW/2008/GII.P7-GII.6/08-AG-1
KM461694	GII.P7	Hu/GII/TW/2011/GII.P7-GII.6/11-FJ-5

The bold letter strains might be estimated the genotypes in this study.

Table S1 (continued). The strains used in this study.

GenBank accession No.	RdRp genotype	Name
KU935739	GII.P7	Hu/GII/CHN/2015/GII.P7-GII.6/0907-26
KX158281	GII.P7	Hu/GII/CA/2015/GII.P7-GII.6/14-55
KX268709	GII.P7	Hu/GII/USA/2014/GII.P7-GII.6/Maryland
KX752057	GII.P7	Hu/GII/CHN/2009/GII.P7-GII.6/Beijing
KY424341	GII.P7	Hu/GII/US/2012/GII.P7-GII.6/BethesdaD1
MF140645	GII.P7	Hu/GII/NL/2013/GII.P7-GII.7/E1300273
MF140676	GII.P7	Hu/GII/NL/2013/GII.P7-GII.6/E1300311
MF140677	GII.P7	Hu/GII/NL/2013/GII.P7-GII.6/E1300312
MG557654	GII.P7	Hu/GII/ETH/2016/GII.P7-GII.6/P3
AB039780	GII.P8	Hu/GII/JP/1998/GII.P8-GII.8/Saitama U25
JX846926	GII.P8	Hu/GII/USA/1988/GII.P8-GII.7/CHDC3936
AB044366	GII.P12	Hu/GII/JP/1999/GII.P12-GII.12/hiroshima
AB220921	GII.P12	Hu/GII/JP/2005/GII.P12-GII.4/Chiba/04-1050
AB220922	GII.P12	Hu/GII/JP/2005/GII.P12-GII.4/Sakai/04-179
AB447448	GII.P12	Hu/GII/JP/2006/GII.P12-GII.4/Sakai2
AF504671	GII.P12	Hu/GII/VNM/2000/GII.P12-GII.10/Vietnam 026
GU980585	GII.P12	Hu/GII/KOR/2006/GII.P12-GII.3/CBNU1
GU991355	GII.P12	Hu/GII/CHN/2009/GII.P12-GII.3/Shanghai/SH312
HM802546	GII.P12	Hu/GII/HKG/2004/GII.P12-GII.4/CU041206
HM802547	GII.P12	Hu/GII/HKG/2004/GII.P12-GII.4/CU041225
HM802548	GII.P12	Hu/GII/HKG/2005/GII.P12-GII.4/CU050128
HM802550	GII.P12	Hu/GII/HKG/2005/GII.P12-GII.4/CU050152
HM802553	GII.P12	Hu/GII/HKG/2005/GII.P12-GII.4/CU051013
HM802555	GII.P12	Hu/GII/HKG/2005/GII.P12-GII.4/CU050130
KF306213	GII.P12	Hu/GII/CHN/2013/GII.P12-GII.3/Jingzhou/2013402
KJ196276	GII.P12	Hu/GII/JP/2002/GII.P12-GII.13/Saitama/T80
KJ196282	GII.P12	Hu/GII/JP/2001/GII.P12-GII.12/Saitama/T15
KJ196294	GII.P12	Hu/GII/JP/2000/GII.P12-GII.12/Saitama/KU16
KY348697	GII.P12	Hu/GII/CHN/2013/GII.P12-GII.3/Guangzhou/GZ2013-L20
KY905334	GII.P12	Hu/GII/AU/2016/GII.P12-GII.3/QLDB207
LC209435	GII.P12	Hu/GII/JP/2004/GII.P12-GII.2/Tochigi-92
KJ196290	GII.P15	Hu/GII/JP/2007/GII.P15-GII.15/Sapporo/HK299
KU954108	GII.P15	Hu/GII/TW/2016/GII.P15-GII.15/Taoyuan/D005
KF895841	GII.P16	Hu/GII/RUS/2012/GII.P16-GII.3/Smolensk/S12-31
KF944110	GII.P16	Hu/GII/RUS/2011/GII.P16-GII.3/Novosibirsk/Nsk-N1659
KJ145322	GII.P16	Hu/GII/TW/2013/GII.P16-GII.13/13-BA-1
KJ196286	GII.P16	Hu/GII/JP/2002/GII.P16-GII.17/Saitama/T87
KJ407074	GII.P16	Hu/GII/USA/2011/GII.P16-GII.2/HS255
KT779557	GII.P16	Hu/GII/RUS/2012/GII.P16-GII.3/Omsk/O1370
KX907727	GII.P16	Hu/GII/USA/2015/GII.P16-GII.4/CA3477
KY421121	GII.P16	Hu/GII/CHN/2016/GII.P16-GII.2/JS1208
KY771081	GII.P16	Hu/GII/HK/2016/GII.P16-GII.2/CUHK-NS-1082
KY865306	GII.P16	Hu/GII/USA/2016/GII.P16-GII.2/Santa Rosa1764
KY865307	GII.P16	Hu/GII/USA/2016/GII.P16-GII.2/Nashville 2122
KY887597	GII.P16	Hu/GII/UK/2016/GII.P16-GII.3/NOR-2604
KY887602	GII.P16	Hu/GII/UK/2015/GII.P16-GII.4/NOR-2516
KY887604	GII.P16	Hu/GII/UK/2015/GII.P16-GII.4/NOR-2518
KY887605	GII.P16	Hu/GII/UK/2015/GII.P16-GII.4/NOR-2520
KY905335	GII.P16	Hu/GII/AU/2016/GII.P16-GII.4/QLDB309
KY905337	GII.P16	Hu/GII/AU/2016/GII.P16-GII.2/Brisbane/QLDB512
KY947548	GII.P16	Hu/GII/USA/2016/GII.P16-GII.13/Carlsbad 4246
KY947549	GII.P16	Hu/GII/USA/2016/GII.P16-GII.4/CS4243
LC145789	GII.P16	Hu/GII/JP/2012/GII.P16-GII.2/Fukui1
LC145795	GII.P16	Hu/GII/JP/2012/GII.P16-GII.2/Hiroshimaciy1
LC145803	GII.P16	Hu/GII/JP/2014/GII.P16-GII.2/Osaka5
LC175468	GII.P16	Hu/GII/JP/2016/GII.P16-GII.4/Kawasaki194
LC209445	GII.P16	Hu/GII/JP/2012/GII.P16-GII.2/Saitama-122
LC209446	GII.P16	Hu/GII/JP/2012/GII.P16-GII.2/Saitama-121

Table S1 (continued). The strains used in this study.

GenBank accession No.	RdRp genotype	Name
LC209454	GII.P16	Hu/GII/JP/2010/GII.P16-GII.2/Osaka-019
LC209459	GII.P16	Hu/GII/JP/2010/GII.P16-GII.2/Kanagawa-51
LC209461	GII.P16	Hu/GII/JP/2009/GII.P16-GII.2/Kanagawa-49
LC209466	GII.P16	Hu/GII/JP/2012/GII.P16-GII.2/Hokkaido-18
LC209477	GII.P16	Hu/GII/JP/2013/GII.P16-GII.2/Ehime-6
LC209480	GII.P16	Hu/GII/JP/2010/GII.P16-GII.2/Ehime-44
LC213889	GII.P16	Hu/GII/JP/2016/GII.P16-GII.2/Ibaraki290
LC213892	GII.P16	Hu/GII/JP/2016/GII.P16-GII.2/Ibaraki374
LC213896	GII.P16	Hu/GII/JP/2016/GII.P16-GII.2/Ibaraki518
LC213898	GII.P16	Hu/GII/JP/2016/GII.P16-GII.2/Ibaraki602
LC213899	GII.P16	Hu/GII/JP/2016/GII.P16-GII.2/Ibaraki607
LC213900	GII.P16	Hu/GII/JP/2016/GII.P16-GII.2/Ibaraki636
LC213901	GII.P16	Hu/GII/JP/2016/GII.P16-GII.2/Ibaraki658
MF167650	GII.P16	Hu/GII/CHN/2017/GII.P16-GII.2/JSCZ201703-19
MF167652	GII.P16	Hu/GII/CHN/2017/GII.P16-GII.2/JSWX201703-10
MG002630	GII.P16	Hu/GII/AU/2017/GII.P16-GII.4/BNE1
MG002631	GII.P16	Hu/GII/AU/2017/GII.P16-GII.4/BNE2
MG002633	GII.P16	Hu/GII/AU/2017/GII.P16-GII.4/BNE4
MG572182	GII.P16	Hu/GII/CHN/2017/GII.P16-GII.1/SDJN170450
MG745987	GII.P16	Hu/GII/CHN/2017/GII.P16-GII.2/GZ78
MG745990	GII.P16	Hu/GII/CHN/2017/GII.P16-GII.2/GZ1528
MG745994	GII.P16	Hu/GII/CHN/2017/GII.P16-GII.2/GZ2113
MG745999	GII.P16	Hu/GII/CHN/2016/GII.P16-GII.2/GZ28626
MG746003	GII.P16	Hu/GII/CHN/2016/GII.P16-GII.2/FJ16235606
MG746008	GII.P16	Hu/GII/CHN/2016/GII.P16-GII.2/CQ031
MG746009	GII.P16	Hu/GII/CHN/2016/GII.P16-GII.2/CQ035
MG746011	GII.P16	Hu/GII/CHN/2017/GII.P16-GII.2/GX170034-1
MG746016	GII.P16	Hu/GII/CHN/2016/GII.P16-GII.2/HNCD2701
MG746024	GII.P16	Hu/GII/CHN/2017/GII.P16-GII.2/HNLD01
MG746025	GII.P16	Hu/GII/CHN/2017/GII.P16-GII.2/LNDL00401
MG746026	GII.P16	Hu/GII/CHN/2017/GII.P16-GII.2/LNLY502
MG746027	GII.P16	Hu/GII/CHN/2016/GII.P16-GII.2/CQ1
MG746031	GII.P16	Hu/GII/CHN/2017/GII.P16-GII.2/LNSY3
MG746034	GII.P16	Hu/GII/CHN/2017/GII.P16-GII.2/SZ127
MG746038	GII.P16	Hu/GII/CHN/2016/GII.P16-GII.2/SZ325
MG746040	GII.P16	Hu/GII/CHN/2016/GII.P16-GII.2/BJHD1608Y123
MG746043	GII.P16	Hu/GII/CHN/2017/GII.P16-GII.2/BJFT463
MG746044	GII.P16	Hu/GII/CHN/2017/GII.P16-GII.2/BJFT640
MG746226	GII.P16	Hu/GII/CHN/2016/GII.P16/BJHDG5
MG746263	GII.P16	Hu/GII/CHN/2016/GII.P16/SZ264
MG746265	GII.P16	Hu/GII/CHN/2016/GII.P16/SZ285
MG746270	GII.P16	Hu/GII/CHN/2016/GII.P16/SZ308
MG746299	GII.P16	Hu/GII/CHN/2016/GII.P16/GZ28486
MG746307	GII.P16	Hu/GII/CHN/2016/GII.P16/GZ28627
MG746313	GII.P16	Hu/GII/CHN/2016/GII.P16/CQ22
MG746372	GII.P16	Hu/GII/CHN/2017/GII.P16/BJFT530
KT780416	GII.P17	Hu/GII/HKG/2015/GII.P17-GII.17/CUHK-NS-657
KT970375	GII.P17	Hu/GII/CHN/2015/GII.P17-GII.17/Guangzhou/GZ2015-L340
KU557788	GII.P17	Hu/GII/CHN/2013/GII.P17-GII.17/2238/GD-JM/2013-08-30
KX356908	GII.P17	Hu/GII/CHN/2015/GII.P17-GII.17/KM1509
KY905332	GII.P17	Hu/GII/AU/2015/GII.P17-GII.17/NSW543Q
LC037415	GII.P17	Hu/GII/JP/2015/GII.P17-GII.17/Kawasaki308
LC043167	GII.P17	Hu/GII/JP/2013/GII.P17-GII.17/Saitama5203
EU424333	GII.P20	Hu/GII/DE/2005/GII.P20-GII.20/Leverkusen267
AB242256	GII.P21	Hu/GII/JP/2003/GII.P21-GII.3/Saga/5424/03
AY845056	GII.P21	Hu/GII/AU/2002/GII.P21-GII.3/C14
AY919139	GII.P21	Hu/GII/AU/2003/GII.P21-GII.1/Picton
EU019230	GII.P21	Hu/GII/IN/2006/GII.P21-GII.21/Ahm/PC03

Table S1 (continued). The strains used in this study.

GenBank accession No.	RdRp genotype	Name
EU921389	GII.P21	Hu/GII/IN/2007/GII.P21-GII.3/Pune/PC52
JN176926	GII.P21	Hu/GII/NL/2006/GII.P21/Nieuwegein004
KJ145323	GII.P21	Hu/GII/TW/2013/GII.P21-GII.3/13-BG-1
KJ196284	GII.P21	Hu/GII/JP/2007/GII.P21-GII.21/Kawasaki/YO284
KM198484	GII.P21	Hu/GII/VNM/2009/GII.P21-GII.3/30212
KM198509	GII.P21	Hu/GII/VNM/2010/GII.P21-GII.3/20479
KM198528	GII.P21	Hu/GII/VNM/2011/GII.P21-GII.3/C2H-25
KM198563	GII.P21	Hu/GII/VNM/2010/GII.P21-GII.3/C2365
KX079488	GII.P21	Hu/GII/KOR/2015/GII.P21-GII.21/JW
MF140685	GII.P21	Hu/GII/NL/2011/GII.P21-GII.3/Rotterdam/E1300323
DQ366347	GII.P22	Hu/GII/JP/2004/GII.P22-GII.2/OsakaNI
KJ196277	GII.P22	Hu/GII/JP/2001/GII.P22-GII.5/Saitama/T49
KM386679	GII.P22	Hu/GII/TW/2007/GII.P22-GII.5/07-AC-1
KM386681	GII.P22	Hu/GII/TW/2011/GII.P22-GII.5/11-FM-1
MG495082	GII.P22	Hu/GII/BD/2012/GII.P22-GII.22/Dhaka1940
MG495083	GII.P22	Hu/GII/BD/2012/GII.P22-GII.25/Dhaka1928
KR232647	GII.P23	Hu/GII/EC/2011/GII.P23-GII.23/Quininde1906
KY496328	GII.P23	Hu/GII/PE/2010/GII.P23-GII.23/PE1848
MG495080	GII.P23	Hu/GII/PE/2011/GII.P23-GII.23/Loreto6422
MG551869	GII.P23	Hu/GII/GT/2012/GII.P23-GII.23/GuatemalaCity3872
KY225989	GII.P24	Hu/GII/PE/2013/GII.P24-GII.24/Loreto1972
MG495084	GII.P24	Hu/GII/US/2013/GII.P24-GII.24/EdenPrairie5457
AY134748	GII.Pc	Hu/GII/US/1976/GII.Pc-GII.2/Snow Mountain
JX846925	GII.Pc	Hu/GII/MYS/1978/GII.Pc-GII.2/KL109
KC597138	GII.Pc	Hu/GII/USA/1975/GII.Pc-GII.2/CHDC2596
KF429769	GII.Pc	Hu/GII/USA/1975/GII.Pc-GII.2/SnowMountRS
AB541323	GII.Pe	Hu/GII/JP/2007/GII.Pe-GII.4/Osaka3
GQ845369	GII.Pe	Hu/GII/AU/2008/GII.Pe-GII.4/Amidale/NSW390I
JX459907	GII.Pe	Hu/GII/AU/2012/GII.Pe-GII.4/Woonona/NSW3309
JX459908	GII.Pe	Hu/GII/AU/2012/GII.Pe-GII.4/Sydney/NSW0514
KC517362	GII.Pe	Hu/GII/TW/2012/GII.Pe-GII.4/Taoyuan/CGMH52
KC517377	GII.Pe	Hu/GII/TW/2012/GII.Pe-GII.4/Taoyuan/CGMH67
KC517378	GII.Pe	Hu/GII/TW/2012/GII.Pe-GII.4/New Taipei/CGMH68
KC577175	GII.Pe	Hu/GII/CHN/2012/GII.Pe-GII.4/Jiangsu2
KF145148	GII.Pe	Hu/GII/JPN/2012/GII.Pe-GII.4/JP10909
KF306214	GII.Pe	Hu/GII/CHN/2013/GII.Pe-GII.4/Jingzhou/2013403
KF509946	GII.Pe	Hu/GII/CA/2012/GII.Pe-GII.4/AlbertaEI063
KJ685406	GII.Pe	Hu/GII/BGD/2012/GII.Pe-GII.4/BG1C0391
KJ955493	GII.Pe	Hu/GII/CHN/2012/GII.Pe-GII.4/Nanshan/OB/12
KP784696	GII.Pe	Hu/GII/ZAF/2012/GII.Pe-GII.4/CapeTown/9772
KT202794	GII.Pe	Hu/GII/CHN/2014/GII.Pe-GII.4/Guangzhou/GZ2014-L106
KT202797	GII.Pe	Hu/GII/CHN/2014/GII.Pe-GII.4/Guangzhou/GZ2014-L295
KT239579	GII.Pe	Hu/GII/AU/2013/GII.Pe-GII.4/NSW028D
KT589391	GII.Pe	Hu/GII/HKG/2015/GII.Pe-GII.17/CUHK-NS-682
KU311158	GII.Pe	Hu/GII/CA/2014/GII.Pe-GII.4/AlbertaEI350
KU678204	GII.Pe	Hu/GII/TW/2016/GII.Pe-GII.4/16-C-2
KX158283	GII.Pe	Hu/GII/CA/2015/GII.Pe-GII.4/15-58
KX657728	GII.Pe	Hu/GII/TW/2016/GII.Pe-GII.4/16-CK-1
KX657731	GII.Pe	Hu/GII/TW/2016/GII.Pe-GII.4/16-FN-1
KX657736	GII.Pe	Hu/GII/TW/2016/GII.Pe-GII.4/D015
KY421039	GII.Pe	Hu/GII/USA/2015/GII.Pe-GII.4/Sydney Variant 2015
KY424328	GII.Pe	Hu/GII/US/2012/GII.Pe-GII.4/RockvilleD1
KY486271	GII.Pe	Hu/GII/USA/2013/GII.Pe-GII.4-Sydney
KY488570	GII.Pe	Hu/GII/TW/2014/GII.Pe-GII.4/D058
KY488572	GII.Pe	Hu/GII/TW/2014/GII.Pe-GII.4/D268
KY488573	GII.Pe	Hu/GII/TW/2014/GII.Pe-GII.4/D088
KY488574	GII.Pe	Hu/GII/TW/2014/GII.Pe-GII.4/D143
KY496327	GII.Pe	Hu/GII/USA/2012/GII.Pe-GII.4/WI2138
KY905333	GII.Pe	Hu/GII/AU/2016/GII.Pe-GII.4/QLDB101

Table S1 (continued). The strains used in this study.

GenBank accession No.	RdRp genotype	Name
LC066046	GII.Pe	Hu/GII/JP/2015/GII.Pe-GII.4/Osaka/OSF78 POL
LC209439	GII.Pe	Hu/GII/JP/2014/GII.Pe-GII.2/Saitama-127
MF140637	GII.Pe	Hu/GII/NL/2013/GII.Pe-GII.4/Rotterdam/E1300285
MF140674	GII.Pe	Hu/GII/NL/2013/GII.Pe-GII.4/Rotterdam/E1300308
MG557655	GII.Pe	Hu/GII/ETH/2016/GII.Pe-GII.10/P4
MF405169	GII.Pf	Hu/GII/US/1971/GII.Pf-GII.2/HenrytonSP17
GQ845370	GII.Pg	Hu/GII/AU/2008/GII.Pg-GII.12/StGeorge/NSW199U
HQ664990	GII.Pg	Hu/GII/USA/2010/GII.Pg-GII.12/HS206
JX846924	GII.Pg	Hu/GII/HKG/1978/GII.Pg-GII.3/HK71
KC597144	GII.Pg	Hu/GII/HKG/1977/GII.Pg-GII.3/HK46
KC597145	GII.Pg	Hu/GII/USA/2010/GII.Pg-GII.12/NIHIC6
**KC962458**	**GII.Pg**	**Hu/GII/ZA/2010/GII.Pg-GII.3/Bushbuckridge6387**
KM198503	GII.Pg	Hu/GII/VNM/2010/GII.Pg-GII.12/C2033
KY442319	GII.Pg	Hu/GII/US/1972/GII.Pg-GII.3/ShippensburgB24
MF668937	GII.Pg	Hu/GII/IDN/2015/GII.Pg-GII.1/ITD11-3
KC576911	GII.Pj	Hu/GII/CAF/1977/GII.Pj/B17
KJ194507	GII.Pm	Hu/GII/NL/1995/GII.Pm-GII.1/Amsterdam/3
M87661	GI.P1	Hu/GI/US/1968/GI.P1-GI.1/Norwalk
HQ392821	GII.P11	Sw/GII/CHN/2009/GII.P11/Ch6
AY823305	GII.P18	Sw/GII/US/2003/GII.P18/OH-QW125
AJ011099	GIII	Bo/GIII/DE/1980/GIII/Jena
NC029647	GIV	Hu/GIV/AU/2010/GIV/LakeMaquarie/NSW2680

The bold letter strains might be estimated the genotypes in this study.

Table S2. Statistical analyses with the multiple comparisons for evolutionary rates in the present norovirus GII *RdRp* strains.

	Norovirus GII	GII.P4	GII.P7	GII.P12	GII.P16	GII.P21	GII.Pe
Norovirus GII		< 2E-16	< 2E-16	< 2E-16	< 2E-16	< 2E-16	< 2E-16
GII.P4	***		< 2E-16	< 2E-16	< 2E-16	6.7E-7	< 2E-16
GII.P7	***	***		< 2E-16	< 2E-16	< 2E-16	< 2E-16
GII.P12	***	***	***		< 2E-16	< 2E-16	< 2E-16
GII.P16	***	***	***	***		< 2E-16	< 2E-16
GII.P21	***	***	***	***	***		< 2E-16
GII.Pe	***	***	***	***	***	***	

The *p*-values and significant signs were shown in the upper and lower sides, respectively.

*** $p<0.001$

Table S3. Statistical analyses with the multiple comparisons for phylogenetic distance in the present norovirus GII *RdRp* strains.

	GII.P4	GII.P7	GII.P12	GII.P16	GII.P21	GII.Pe
GII.P4		< 2E-16	1.8E-15	< 2E-16	3.6E-11	< 2E-16
GII.P7	***		< 2E-16	< 2E-16	< 2E-16	< 2E-16
GII.P12	***	***		2.8E-2	5.9E-1	1.2E-15
GII.P16	***	***	*		2.7E-2	< 2E-16
GII.P21	***	***		*		2.7E-13
GII.Pe	***	***	***	***	***	

The *p*-values and significant signs were shown in the upper and lower sides, respectively.

* $p < 0.05$, *** $p < 0.001$

Table S4. The substitution sites and the negative selection sites of norovirus GII strains.

Lineage	Genotype	Accession No.	2	3	4	5	15	16	18	33	34	35	49	54	55	60	65	69	72	76	77	79	81	82	84	85	88
1	GII.P8	AB039780	G	E	D	H	A	P	K	P	D	S	K	E	K	Q	D	P	E	K	P	R	S	V	E	E	K
	GII.P6	AB039778	N	•	S	L	G	•	•	•	•	A	•	•	•	•	•	•	•	•	•	•	T	•	•	•	•
	GII.P7	AB039777	•	•	N	•	•	•	•	•	•	•	•	•	•	•	•	•	•	•	•	•	A	•	•	•	•
	GII.P15	KU954108	G	•	D	K	•	L	•	•	•	•	•	•	•	•	E	•	•	•	•	K	N	•	D	•	•
	GII.P20	EU424333	•	D	•	•	S	V	•	•	E	•	•	•	•	H	D	•	•	•	•	N	T	•	E	•	•
2	GII.P1	U07611	•	•	•	•	G	P	S	T	T	P	•	K	S	Q	•	•	•	•	Q	K	S	•	•	A	•
	GII.P2	DQ456824	•	•	N	•	•	•	N	•	•	•	•	•	G	•	•	•	•	•	P	•	•	•	•	•	•
	GII.P3	KJ194500	•	•	G	•	•	•	•	N	A	•	•	•	•	•	•	S	•	•	•	N	•	•	•	S	•
	GII.P4	AB541272	•	•	N	•	•	•	S	T	•	•	•	•	•	•	•	P	•	R	•	K	•	•	•	A	•
	GII.P5	KJ196288	•	•	D	•	•	•	G	N	T	•	R	•	•	•	•	•	•	K	•	•	•	•	•	•	Q
	GII.P12	AB220922	•	•	S	•	•	•	S	T	A	•	K	•	•	•	•	•	•	•	•	•	•	•	•	•	K
	GII.P16	KJ196286	•	•	N	•	•	•	G	N	T	•	R	•	•	•	•	•	•	•	•	•	N	•	•	•	Q
	GII.P17	LC037415	•	•	•	•	•	•	N	•	A	•	•	•	•	•	•	•	•	•	•	N	•	•	•	S	K
	GII.P21	AY919139	•	•	•	•	•	•	•	T	T	•	K	•	•	•	•	•	•	•	•	K	S	•	•	A	•
	GII.Pc	AY134748	•	•	•	•	•	•	•	•	V	•	•	•	•	•	•	•	•	•	•	•	•	•	•	•	•
	GII.Pe	JX459907	•	•	S	•	•	•	S	•	T	•	•	•	•	•	•	•	•	•	•	R	N	•	•	•	•
	GII.Pf	MF405169	•	•	N	•	•	•	•	•	•	•	•	•	•	•	•	•	•	•	•	•	S	•	D	•	•
	GII.Pg	GQ845370	•	N	•	•	•	•	N	•	A	•	•	•	•	•	•	•	•	•	•	K	•	•	E	•	•
	GII.Pj	KC576911	•	D	•	•	•	•	S	•	T	•	•	•	•	•	•	•	•	•	•	•	•	•	•	•	•
	GII.Pm	KJ194507	A	•	•	•	•	•	•	•	•	•	•	•	•	•	•	•	•	•	Q	R	N	•	•	•	R
3	GII.P22	KJ196277	G	•	•	H	•	•	•	N	A	•	•	•	•	•	E	•	A	•	P	•	A	I	•	•	E
	GII.P23	MG495080	•	•	•	•	•	•	N	•	T	•	•	•	•	•	•	•	•	•	•	•	•	V	•	•	•
	GII.P24	KY225989	•	•	•	•	•	•	•	•	•	•	•	•	•	•	•	•	•	•	•	•	•	I	•	•	•

The squares of negative selection sites are coloured green.

Table S4 (continued). The substitution sites and the negative selection sites of norovirus GII strains.

Lineage	Genotype	Accession No.	90	91	94	97	98	99	101	102	103	105	106	107	121	125	126	129	130	132	134	135	137	139	151	153	154
1	GII.P8	AB039780	V	M	L	T	I	E	A	K	P	T	Y	S	S	V	R	E	H	N	E	S	T	P	Y	E	G
	GII.P6	AB039778	•	•	•	•	•	D	•	•	•	•	•	•	•	I	•	D	•	•	•	•	•	•	•	Q	A
	GII.P7	AB039777	•	•	•	•	•	•	•	•	•	•	•	•	•	•	K	•	•	•	•	•	•	•	•	•	**•**
	GII.P15	KU954108	•	•	•	•	•	N	•	•	•	S	•	•	•	L	R	•	•	•	•	•	•	•	•	•	•
	GII.P20	EU424333	•	•	•	•	•	D	•	•	•	T	•	A	•	•	K	•	•	•	D	•	•	•	•	•	•
2	GII.P1	U07611	**I**	I	•	•	•	•	P	Q	K	S	F	•	H	I	R	•	C	•	•	•	•	K	F	E	G
	GII.P2	DQ456824	•	•	•	•	•	•	•	•	•	•	Y	•	Y	V	•	E	H	•	E	•	•	•	•	•	•
	GII.P3	KJ194500	•	•	•	V	•	•	•	•	•	•	•	•	H	•	•	D	Y	S	•	•	•	•	Y	•	•
	GII.P4	AB541272	•	**•**	•	T	•	**•**	•	E	**•**	•	**F**	•	**•**	M	•	**•**	C	N	•	•	•	**•**	**F**	•	•
	GII.P5	KJ196288	•	•	•	•	L	•	•	Q	•	T	Y	•	•	T	•	E	F	•	•	T	•	•	•	•	•
	GII.P12	AB220922	•	V	•	•	I	•	•	•	•	S	F	•	•	M	•	D	C	•	•	S	•	•	•	•	•
	GII.P16	KJ196286	•	I	•	•	L	**•**	•	•	•	T	Y	•	•	V	•	E	F	•	•	T	V	•	•	•	•
	GII.P17	LC037415	•	•	•	•	I	•	•	•	•	S	•	•	Y	•	•	D	Y	S	•	S	T	•	Y	•	•
	GII.P21	AY919139	•	•	I	•	•	•	•	•	•	T	•	•	•	M	•	E	C	N	•	T	•	•	•	•	•
	GII.Pc	AY134748	•	•	L	•	•	•	•	•	•	S	F	S	H	I	•	D	•	•	•	S	•	•	•	•	•
	GII.Pe	JX459907	•	•	•	•	•	**•**	•	•	•	•	•	A	•	M	•	•	•	•	•	•	•	•	F	•	•
	GII.Pf	MF405169	•	•	•	•	•	•	•	•	•	T	Y	•	F	V	•	E	H	•	•	T	•	•	•	•	•
	GII.Pg	GQ845370	•	•	•	•	•	•	•	•	•	•	F	•	Y	•	•	•	•	•	•	S	•	•	•	•	•
	GII.Pj	KC576911	•	•	•	•	•	•	•	•	•	S	•	•	H	I	•	D	C	•	•	•	•	•	•	•	•
	GII.Pm	KJ194507	•	•	•	•	•	•	•	•	•	T	•	•	•	•	•	•	•	•	D	•	•	•	Y	•	•
3	GII.P22	KJ196277	•	C	•	I	•	•	•	•	•	S	Y	•	•	M	K	V	N	•	E	•	•	V	F	Q	•
	GII.P23	MG495080	•	•	•	T	•	E	•	•	•	•	•	•	•	K	•	D	•	•	•	•	•	•	Y	•	•
	GII.P24	KY225989	•	•	•	•	•	•	•	•	•	•	•	•	•	•	•	•	•	•	•	•	•	•	F	•	•

The squares of negative selection sites are coloured green.

Table S4 (continued). The substitution sites and the negative selection sites of norovirus GII strains.

Lineage	Genotype	Accession No.	156	157	158	160	163	173	175	177	178	189	190	193	198	203	204	205	206	208	209	211	212	215	221	227	228
1	GII.P8	AB039780	H	M	N	M	A	D	I	K	K	L	G	L	A	L	D	S	M	E	S	I	S	C	I	L	I
	GII.P6	AB039778	•	•	Q	V	•	•	•	•	•	•	•	V	•	M	•	•	•	A	•	•	A	•	M	I	•
	GII.P7	AB039777	•	V	•	•	•	•	•	•	•	•	•	I	•	•	•	•	•	•	•	•	•	•	•	•	•
	GII.P15	KU954108	•	M	S	•	•	•	•	•	•	•	•	V	•	•	•	•	•	E	•	V	M	•	•	•	•
	GII.P20	EU424333	•	V	•	•	•	•	•	•	•	•	•	•	•	L	•	•	•	•	•	•	A	•	•	•	•
2	GII.P1	U07611	N	M	T	•	G	•	•	G	•	•	A	I	•	M	•	E	L	A	H	•	T	V	•	•	•
	GII.P2	DQ456824	H	•	•	•	A	E	V	•	•	•	•	V	S	•	•	•	•	•	•	•	•	I	•	•	V
	GII.P3	KJ194500	•	•	P	•	•	D	I	•	•	•	S	I	A	•	•	•	•	•	N	I	•	V	•	•	I
	GII.P4	AB541272	N	•	T	•	•	•	•	•	•	•	A	•	•	•	•	•	•	•	H	V	•	I	•	•	•
	GII.P5	KJ196288	H	•	•	•	•	•	•	•	•	•	S	V	•	•	E	•	M	•	•	I	S	V	•	•	•
	GII.P12	AB220922	N	•	•	•	G	•	•	•	•	•	A	I	•	•	•	•	L	•	•	V	T	•	•	•	•
	GII.P16	KJ196286	H	•	•	•	A	•	•	•	•	•	S	•	•	•	D	•	M	•	•	I	S	•	•	•	•
	GII.P17	LC037415	•	•	Q	•	•	•	•	•	•	•	•	•	•	•	•	•	F	•	N	•	T	I	•	•	•
	GII.P21	AY919139	N	•	T	•	G	•	•	•	•	•	•	•	•	•	•	•	L	•	H	V	•	V	•	•	•
	GII.Pc	AY134748	•	•	•	•	•	•	•	•	Q	•	A	•	•	•	•	•	•	•	•	•	•	•	•	•	•
	GII.Pe	JX459907	•	•	•	•	•	•	V	•	K	•	•	•	•	•	•	•	•	•	•	•	•	•	•	•	•
	GII.Pf	MF405169	H	•	•	•	A	•	•	•	•	•	•	V	S	•	•	•	•	S	•	•	•	I	•	•	V
	GII.Pg	GQ845370	•	•	•	•	G	•	I	•	•	•	•	I	A	•	•	•	•	A	•	•	•	•	•	•	I
	GII.Pj	KC576911	S	•	•	•	•	•	•	•	•	•	•	•	•	•	•	•	•	•	•	•	•	V	•	•	•
	GII.Pm	KJ194507	N	•	•	•	•	E	•	•	T	•	•	•	•	•	E	•	•	•	•	•	•	I	•	•	•
3	GII.P22	KJ196277	H	•	Q	•	A	S	•	E	K	V	G	•	•	•	D	•	M	•	N	•	N	•	•	•	•
	GII.P23	MG495080	•	•	•	•	G	•	•	•	T	L	•	•	S	•	•	•	•	•	•	•	•	V	•	•	•
	GII.P24	KY225989	•	•	•	•	•	•	•	•	N	•	•	•	•	•	•	•	•	•	•	•	R	•	•	•	•

The squares of negative selection sites are coloured green.

Table S4 (continued). The substitution sites and the negative selection sites of norovirus GII strains.

Lineage	Genotype	Accession No.	230	231	233	234	235	236	237	253	254	256	259	260	261	264	265	267	270	272	274	276	281	284	285	291	292
1	GII.P8	AB039780	E	K	S	K	Y	T	Y	A	V	S	M	E	V	K	F	A	E	A	V	A	A	R	L	V	I
	GII.P6	AB039778	D	•	•	•	•	R	•	S	I	•	•	•	•	R	•	•	•	•	•	•	•	Q	•	•	•
	GII.P7	AB039777	•	•	•	•	•	•	•	•	•	•	•	•	•	•	•	•	•	•	•	•	•	•	•	•	•
	GII.P15	KU954108	•	•	A	•	•	K	•	•	•	•	•	•	•	K	•	•	•	•	•	•	•	•	•	•	V
	GII.P20	EU424333	•	•	S	•	•	R	F	C	•	A	•	•	•	•	•	•	•	•	•	•	•	•	•	•	I
2	GII.P1	U07611	E	•	•	R	•	K	Y	A	V	•	L	•	I	•	•	P	H	•	•	•	S	V	M	K	•
	GII.P2	DQ456824	•	•	•	•	•	R	•	•	•	•	M	•	•	•	•	A	Q	•	I	•	•	•	V	•	•
	GII.P3	KJ194500	•	•	•	•	•	•	•	•	•	•	L	•	•	R	•	•	•	•	•	•	A	•	•	•	•
	GII.P4	AB541272	•	R	•	•	•	T	•	•	•	•	•	•	•	K	•	P	H	•	V	•	S	•	•	T	•
	GII.P5	KJ196288	•	K	•	•	•	K	•	•	•	•	M	•	•	R	•	A	Q	S	K	•	A	•	•	K	•
	GII.P12	AB220922	•	R	•	•	•	•	•	•	•	•	L	•	•	K	•	P	H	A	•	•	S	•	M	•	•
	GII.P16	KJ196286	•	K	•	•	•	•	•	•	•	•	•	•	•	R	•	A	Q	•	T	•	A	•	V	•	•
	GII.P17	LC037415	•	•	•	•	•	R	•	•	•	E	•	•	•	•	•	•	•	•	I	•	S	•	•	•	•
	GII.P21	AY919139	•	•	•	•	•	•	•	•	•	V	M	•	•	K	•	S	H	•	V	•	•	•	M	•	•
	GII.Pc	AY134748	•	•	•	•	F	S	•	•	•	A	L	•	•	•	•	P	•	•	I	•	A	•	•	•	•
	GII.Pe	JX459907	•	•	•	•	Y	R	•	D	•	•	•	•	•	•	•	•	•	•	•	•	S	•	•	Q	•
	GII.Pf	MF405169	•	•	•	•	•	•	•	A	•	•	M	•	•	•	•	A	Q	•	V	•	•	•	V	K	•
	GII.Pg	GQ845370	•	•	•	•	•	T	•	•	•	•	L	•	•	•	•	P	H	•	I	•	•	•	M	•	•
	GII.Pj	KC576911	•	R	•	•	•	R	•	•	•	•	•	•	•	•	•	•	•	•	•	•	•	•	•	•	•
	GII.Pm	KJ194507	•	•	•	•	•	K	•	•	I	•	•	•	•	•	•	S	•	•	K	•	•	•	•	•	•
3	GII.P22	KJ196277	•	K	•	K	•	•	•	H	•	G	•	•	•	R	•	A	E	•	I	S	A	•	V	•	•
	GII.P23	MG495080	•	•	•	•	•	R	•	C	V	•	•	D	•	•	Y	•	•	•	•	A	S	•	M	•	•
	GII.P24	KY225989	•	•	•	•	•	•	•	Y	•	•	•	E	•	•	F	•	•	•	•	•	•	•	•	•	•

The squares of negative selection sites are coloured green.

Table S4 (continued). The substitution sites and the negative selection sites of norovirus GII strains.

Lineage	Genotype	Accession No.	293	294	295	312	315	316	319	321	322	324	325	326	328	330	331	332	334	335	337	338	352	356	360	363	368
1	GII.P8	AB039780	S	V	Q	A	I	L	S	M	S	V	S	G	S	E	V	V	A	N	C	F	N	E	R	R	V
	GII.P6	AB039778	•	•	•	•	•	•	•	•	A	•	•	•	•	D	•	•	•	H	•	•	•	S	Q	•	•
	GII.P7	AB039777	•	•	•	•	•	•	•	•	•	•	•	•	•	•	•	•	•	•	•	•	•	M	•	•	•
	GII.P15	KU954108	•	•	•	•	•	I	•	•	•	•	•	•	•	•	•	I	•	•	•	•	•	A	L	•	•
	GII.P20	EU424333	•	•	•	•	•	•	•	•	•	•	•	•	•	E	•	•	•	N	•	•	•	•	Q	•	•
2	GII.P1	U07611	•	I	N	T	L	L	C	L	S	•	T	D	•	D	I	•	•	•	L	•	K	E	A	K	K
	GII.P2	DQ456824	•	•	•	A	•	•	•	•	•	•	•	•	•	•	•	V	•	•	•	•	•	•	G	R	•
	GII.P3	KJ194500	T	•	•	•	•	•	•	•	•	•	•	G	G	•	•	I	•	•	M	Y	•	•	A	K	R
	GII.P4	AB541272	S	•	•	•	•	•	•	•	•	•	•	N	S	•	T	•	•	•	L	F	•	•	G	R	K
	GII.P5	KJ196288	•	•	•	T	•	•	•	•	•	T	•	•	•	•	I	•	•	•	M	•	•	•	T	•	•
	GII.P12	AB220922	•	•	•	A	•	•	•	•	•	V	•	•	•	•	•	•	•	•	L	•	•	•	A	K	•
	GII.P16	KJ196286	•	•	•	T	•	•	•	•	•	•	•	•	•	•	•	V	•	•	M	•	•	•	T	R	•
	GII.P17	LC037415	A	•	•	A	•	•	•	•	•	•	•	G	G	•	•	I	•	•	•	Y	•	•	A	K	•
	GII.P21	AY919139	S	•	•	•	•	•	•	•	•	•	•	D	S	•	•	•	•	•	L	F	•	•	T	•	•
	GII.Pc	AY134748	T	•	•	•	•	•	•	•	•	•	•	N	A	•	•	•	•	•	•	•	•	•	A	•	•
	GII.Pe	JX459907	S	•	S	•	•	•	•	•	•	•	•	D	S	•	•	•	•	•	•	•	•	•	•	•	•
	GII.Pf	MF405169	•	•	N	•	•	•	•	•	•	•	•	•	•	•	•	V	•	•	•	•	•	G	E	•	•
	GII.Pg	GQ845370	•	•	T	•	•	•	•	•	•	•	•	•	•	•	•	I	•	•	•	•	•	E	A	•	•
	GII.Pj	KC576911	•	•	N	•	•	•	•	•	•	•	•	N	•	•	•	•	•	•	•	•	•	•	•	•	•
	GII.Pm	KJ194507	•	•	•	•	•	•	•	•	•	•	•	D	•	•	•	•	•	•	•	•	•	•	•	R	•
3	GII.P22	KJ196277	T	•	•	S	•	I	•	I	•	I	S	G	•	•	V	V	T	•	C	•	•	M	N	K	I
	GII.P23	MG495080	•	•	•	A	•	L	•	M	•	V	•	•	•	•	•	•	S	•	•	•	•	Q	D	R	•
	GII.P24	KY225989	•	•	•	•	•	•	•	•	A	•	•	•	•	•	•	I	•	•	•	•	•	•	•	K	•

The squares of negative selection sites are coloured green.

Table S4 (continued). The substitution sites and the negative selection sites of norovirus GII strains.

Lineage	Genotype	Accession No.	380	381	382	383	384	386	394	395	396	400	403	405	407	408	409	412	415	416	419	421	424	425	427	429	431
1	GII.P8	AB039780	V	I	T	Q	D	N	T	I	V	A	F	K	D	R	D	L	L	Y	R	P	E	N	F	S	I
	GII.P6	AB039778	•	•	•	E	•	T	S	•	A	•	•	•	•	Q	•	•	•	•	•	•	•	•	Y	•	V
	GII.P7	AB039777	•	•	•	•	N	•	•	•	•	•	•	•	•	•	•	•	•	•	•	•	•	•	•	•	•
	GII.P15	KU954108	•	•	•	•	D	H	H	•	T	•	•	•	•	•	•	•	•	•	•	•	•	•	•	C	•
	GII.P20	EU424333	•	•	•	•	N	•	T	V	•	•	•	•	•	•	•	•	•	•	•	•	•	•	•	S	•
2	GII.P1	U07611	I	•	S	•	D	D	•	•	•	•	•	•	E	•	S	•	M	•	•	•	•	D	S	T	I
	GII.P2	DQ456824	•	•	•	•	•	N	•	•	•	•	•	•	D	•	•	•	L	•	K	•	•	•	F	S	•
	GII.P3	KJ194500	V	•	•	•	•	•	•	•	•	•	•	•	E	•	•	•	•	•	R	•	•	•	S	T	•
	GII.P4	AB541272	•	•	•	•	•	•	•	•	•	•	•	•	•	•	•	•	M	•	•	S	•	•	•	•	•
	GII.P5	KJ196288	I	•	•	•	•	D	•	•	S	•	•	•	D	•	•	•	•	•	•	P	•	•	N	•	•
	GII.P12	AB220922	V	•	•	•	•	N	•	•	T	•	•	•	E	•	•	•	•	•	•	•	•	•	S	•	•
	GII.P16	KJ196286	I	•	•	•	•	•	•	•	S	•	•	•	D	•	•	•	•	•	•	•	•	•	N	•	•
	GII.P17	LC037415	V	•	•	•	•	•	•	•	T	•	•	•	•	•	N	•	L	•	•	•	•	•	S	•	•
	GII.P21	AY919139	I	•	•	•	•	D	•	•	•	•	•	•	•	•	S	•	M	•	•	•	•	•	F	•	•
	GII.Pc	AY134748	•	•	•	•	•	N	•	•	•	•	•	•	•	•	•	•	I	•	•	•	•	•	S	•	•
	GII.Pe	JX459907	V	•	•	•	•	D	•	•	•	•	•	•	E	•	•	•	M	•	•	•	•	•	F	•	•
	GII.Pf	MF405169	I	•	•	•	•	N	•	•	•	•	•	R	D	•	•	•	L	•	K	•	•	•	•	S	•
	GII.Pg	GQ845370	V	•	•	•	•	D	•	•	•	•	•	K	E	•	•	•	M	•	R	•	•	•	A	T	•
	GII.Pj	KC576911	I	•	•	•	•	N	•	•	•	•	•	•	•	•	•	•	•	•	•	•	•	•	S	•	•
	GII.Pm	KJ194507	•	•	•	•	•	D	•	•	•	•	•	•	D	•	•	•	•	•	•	•	•	•	•	•	•
3	GII.P22	KJ196277	•	V	K	•	N	•	D	•	C	•	Y	•	•	•	•	M	L	F	K	•	D	•	N	•	•
	GII.P23	MG495080	•	I	R	•	S	E	•	•	•	•	•	•	•	•	•	•	•	Y	•	•	E	•	•	•	•
	GII.P24	KY225989	•	•	K	•	•	•	•	•	•	V	F	•	•	•	•	•	•	•	R	•	•	•	S	•	•

The squares of negative selection sites are coloured green.

Table S4 (continued). The substitution sites and the negative selection sites of norovirus GII strains.

Lineage	Genotype	Accession No.	434	437	438	442	447	448	451	453	456	457	458	461	464	466	468	469	471	472	480	502	503
1	GII.P8	AB039780	S	A	T	A	A	S	G	Q	K	K	V	M	N	I	S	G	L	E	A	E	N
	GII.P6	AB039778	•	•	•	•	•	•	•	•	•	•	•	•	S	•	•	•	•	•	•	•	S
	GII.P7	AB039777	•	•	•	•	•	•	•	•	•	•	•	•	N	•	•	•	•	•	•	•	G
	GII.P15	KU954108	•	•	•	•	•	•	•	•	•	•	•	•	•	•	•	•	•	•	•	D	•
	GII.P20	EU424333	•	•	•	•	•	•	•	•	•	•	•	•	S	•	•	•	•	•	•	•	•
2	GII.P1	U07611	•	P	I	S	•	A	•	A	S	•	I	L	A	L	E	•	M	D	P	S	F
	GII.P2	DQ456824	•	•	•	•	•	•	E	S	•	•	•	•	S	•	•	S	•	•	•	•	L
	GII.P3	KJ194500	A	•	V	A	S	S	G	•	•	•	V	•	•	•	•	G	•	•	S	•	F
	GII.P4	AB541272	S	•	I	S	A	A	•	A	•	•	I	•	A	•	•	•	•	•	P	•	•
	GII.P5	KJ196288	•	•	•	A	•	S	•	S	•	•	•	•	T	•	•	•	•	•	•	•	•
	GII.P12	AB220922	•	•	•	S	•	A	•	A	•	•	•	•	A	•	•	•	•	•	•	•	•
	GII.P16	KJ196286	•	•	•	A	•	S	•	S	•	•	•	•	T	•	•	•	•	•	•	•	•
	GII.P17	LC037415	A	•	V	•	S	•	•	•	•	•	V	•	S	•	•	•	•	•	S	•	•
	GII.P21	AY919139	S	•	I	S	A	A	•	T	•	•	I	•	•	•	•	•	•	•	P	•	S
	GII.Pc	AY134748	•	•	•	•	•	•	•	•	T	•	•	•	T	•	•	•	•	•	•	•	F
	GII.Pe	JX459907	•	•	•	•	•	•	•	A	S	•	•	•	A	•	•	•	•	•	•	•	•
	GII.Pf	MF405169	•	•	•	•	•	•	E	S	•	•	•	•	S	•	•	•	•	•	•	•	L
	GII.Pg	GQ845370	•	•	•	•	•	•	G	•	•	•	•	•	•	•	•	•	•	•	•	N	F
	GII.Pj	KC576911	•	•	•	•	•	•	•	A	•	•	•	•	T	•	•	•	•	•	•	S	•
	GII.Pm	KJ194507	•	•	•	•	•	•	•	•	•	•	•	•	A	•	•	•	•	•	•	•	•
3	GII.P22	KJ196277	•	•	•	•	•	•	•	•	K	•	V	•	T	•	•	•	•	•	•	E	G
	GII.P23	MG495080	•	•	V	•	•	•	•	T	•	R	•	•	•	•	•	•	•	•	•	•	S
	GII.P24	KY225989	•	•	•	•	•	•	•	A	•	K	•	•	•	•	•	•	•	•	•	•	G

The squares of negative selection sites are coloured green.

Table S5. Parameters for evolutionary rates and Bayesian skyline plot analyses in norovirus GII *RdRp* regions.

**Dataset**	**Number of strains**	**Substitution model**	**Clock model**	**Tree prior model**	**Length of MCMC chain**	**Log parameter**
Norovirus GII	484	GTR+Γ+I	Relaxed Clock Log Normal	Coalescent Constant Population	150,000,000	3,000
			Strict Clock	Coalescent Bayesian Skyline	150,000,000	30,000
GII.P4	248	GTR+Γ+I	Relaxed Clock Log Normal	Coalescent Exponential Population	100,000,000	2,000
			Relaxed Clock Log Normal	Coalescent Bayesian Skyline	200,000,000	100,000
GII.P7	28	TIM2ef+Γ	Relaxed Clock Exponential	Coalescent Exponential Population	50,000,000	1,000
			Relaxed Clock Log Normal	Coalescent Bayesian Skyline	50,000,000	2,000
GII.P12	20	TrN+I	Relaxed Clock Exponential	Coalescent Exponential Population	50,000,000	1,000
			Relaxed Clock Log Normal	Coalescent Bayesian Skyline	50,000,000	2,000
GII.P16	71	TrNef+Γ+I	Relaxed Clock Exponential	Coalescent Exponential Population	50,000,000	1,000
			Relaxed Clock Log Normal	Coalescent Bayesian Skyline	100,000,000	10,000
GII.P21	14	GTR+Γ	Strict Clock	Coalescent Constant Population	50,000,000	1,000
			Relaxed Clock Log Normal	Coalescent Bayesian Skyline	60,000,000	1,000
GII.Pe	38	K80+Γ	Relaxed Clock Log Normal	Coalescent Constant Population	50,000,000	1,000
			Relaxed Clock Log Normal	Coalescent Bayesian Skyline	50,000,000	4,000

Parameters for evolutionary rates and BSP are indicated in upper and lower lines, respectively.
